# A Hybrid N‐AHP‐Based TOPSIS Decision Support Approach for Investigation of the Effect of Different Solvents on the Bioactive Properties, Anticancer, and Antimicrobial Activities of *Aronia melanocarpa* Extract

**DOI:** 10.1002/fsn3.70122

**Published:** 2025-03-30

**Authors:** Gulsum Ucak Ozkaya

**Affiliations:** ^1^ Scientific Research Projects Coordination Unit Mimar Sinan Art Fine University Istanbul Turkey

**Keywords:** antimicrobial activities, antioxidant activities, *Aronia melanocarpa*, bioactive component, cytotoxic activities, N‐AHP‐based TOPSIS

## Abstract

The aim of this study was to extract the bioactive components in *Aronia melanocarpa* L. fruit, commonly referred to as chokeberry, using various solvents (80% methanol + 1% formic acid, 80% ethanol, and 70% acetone) individually and to evaluate them through the Neutrosophic‐Analytical Hierarchy Process (N‐AHP)‐based TOPSIS method. The ethanolic (EEA), methanolic (MEA), and acetone (AEA) extracts derived from Aronia fruit were analyzed for total phenolic, flavonoid, and anthocyanin contents, as well as antioxidant, antimicrobial, and cytotoxic activities. The EEA exhibited the highest overall anthocyanin content. The MEA exhibited the highest DPPH levels. The AEA exhibited the highest levels of total phenolic compounds, total flavonoid content, and CUPRAC values. The EEA, MEA, and AEA exhibited IC50 values of 11.65, 11.71, and 10.51 mg/mL against Caco‐2 adenocarcinoma cells, respectively. EEA and MEA exhibited superior antimicrobial efficacy compared to AEA. According to the weights determined by N‐AHP, the extraction with methanol was found to be the best extraction method according to the TOPSIS analysis results performed on real data. In other words, the extract with the lowest IC50 value and the highest antimicrobial activity was the one extracted with methanol. This study concluded that the N‐AHP‐based TOPSIS method is a significant multiple decision‐making approach for evaluating plant extracts based on established criteria and is applicable to food and phytochemical sciences.

## Graphical abstract

This study employed the N‐AHP‐based TOPSIS method to assess the alternatives, utilizing criteria weightings established through expert opinion. For the first time in the field of food science, extracts obtained from Aronia fruits were sequenced using N‐AHP‐based TOPSIS. Upon evaluating the data alongside every criterion, it was found that the methanol‐extracted Aronia fruit was the superior extract based on the weighting established by the expert's opinion.

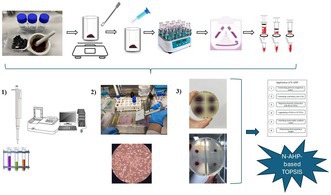

## 1 Introduction

*Aronia melanocarpa* is a shrub species classified under the Rosaceae family, originating from North America (Aydogdu Emir et al. 2023). It has been identified as a novel food resource, as officially declared by the National Health Commission (Chen et al. 2023) and commonly known as chokeberry, and comprises three species: *A. arbutifolia* (L.) Pers. (red chokeberry), *A. melanocarpa* (Michx.) Elliot (black chokeberry), and *A. prunifolia* (Marshall) Rehder (purple chokeberry) (Oszmiański and Lachowicz 2016). The black chokeberry has garnered attention due to its status as one of the most abundant sources of bioactive chemicals, exhibiting significant biological and nutritional significance (Platonova et al. 2021). Vitamins, beta‐carotene, dietary fiber, minerals, and organic acids are all present in good amounts in black chokeberry (Kulling and Rawel 2008).

Black chokeberry is widely acknowledged as a highly abundant reservoir of antioxidants, exhibiting superior antioxidant content when compared to currants, cranberries, blueberries, elderberries, and gooseberries (Brand 2010). Nonetheless, due to the tart flavor profile exhibited by berries, fresh black chokeberries are not widely favored among consumers. Therefore, they are typically consumed in various processed forms such as juices, nectars, syrups, jams, preserves, wines, tinctures, desserts, jellies, teas, and dietary supplements (Mayer‐Miebach et al. 2012). In addition to these food products, currently, studies are being conducted on the utilization of Aronia fruits and their by‐products in the production of functional products since Aronia fruits have a significant potential for antioxidants due to their high polyphenol content. Recent studies have suggested the potential utilization of chokeberry pomace extract as a supplementary ingredient in apple juice, thereby aiding in its preservation and exerting a favorable influence on its color and flavor attributes owing to the presence of antioxidant pigments (Jara‐Palacios et al. 2019; Piasecka et al. 2022; Šic Žlabur et al. 2017). Based on a research investigation, the incorporation of freeze‐dried and powdered chokeberry pomace into white bread resulted in a notable augmentation of its antioxidant activity and phenolic content (Cacak‐Pietrzak et al. 2023). In a particular study, chokeberry juice was employed as an ingredient to create a beverage based on wort. The application of chokeberry juice at various doses resulted in enhanced sensory attributes of the wort (Habschied et al. 2023). A study was undertaken by Du and Myracle (2018) to investigate the impact of incorporating Aronia and elderberry into kefir drinks with the aim of enhancing the bioactive component content. Upon conducting phytochemical tests, it was shown that the inclusion of Aronia juice in kefir led to an increase in both phenolic components and anthocyanin levels.

The utilization of black chokeberry is commonly observed in light of its advantageous effects on health, which can be attributed to the abundance of bioactive chemicals present in its fruits (Kasprzak‐Drozd et al. 2021). The fruits are widely recognized for their significant capacity to scavenge free radicals, which can be attributed to their elevated levels of procyanidins, anthocyanins, flavonoids, and phenolic acids (Cvetanović et al. 2018). With these compounds, the black chokeberry demonstrates various beneficial properties, including antioxidant, anti‐inflammatory, antitumoral, antibacterial, antifungal, anti‐diabetic, and anti‐obesity actions. Additionally, it has shown a potential to protect the liver, heart, and nervous system (Negreanu‐Pirjol et al. 2023).

Considering the positive contribution of black chokeberry to health in light of the information provided, it is of great importance to extract purposeful bioactive substances from the fruit. Therefore, within the scope of our investigation, an extraction procedure was conducted to find optimum solvents employing ethanol, methanol, and acetone as solvents, and the bioactive characteristics, anticancer effects, and antibacterial and antifungal activities of the Aronia extracts were investigated. Numerous studies in the literature investigate the impact of independent variables on the response for solvent optimization (Lee et al. 2024; Lin et al. 2022; Vázquez‐Espinosa et al. 2019). Response surface methodology is the most commonly employed technique (Simić et al. 2016). Moreover, the multi‐criteria decision‐making (MCDM) methods are employed to assess the responses derived from various independent and dependent variables (Cebi et al. 2019; Vural et al. 2021). This work seeks to assess the empirical data acquired through extraction with methanol, ethanol, and acetone and to provide an extensive analysis utilizing the neutrosophic AHP (N‐AHP)‐based TOPSIS method, a multi‐criteria decision‐making approach, to identify the most suitable response. The neutrosophic AHP‐based TOPSIS technique is an effective tool for decision‐making procedures characterized by significant uncertainty and ambiguity (Nabeeh et al. 2019). This approach enables the concurrent assessment of numerous criteria (Sánchez‐Garrido et al. 2021). The neutrosophic AHP‐based TOPSIS method enables the determination of the most suitable alternative by establishing a balance between different criteria (Akbaş et al. 2024). This enables decision‐makers to make more informed and strategic choices.

Ultimately, three academics from the department of food engineering, specializing in functional foods, food chemistry, food microbiology, and food biotechnology, were questioned to get their assessments and perspectives regarding the criteria for black chokeberry extraction. The data collected based on expert opinions was assessed using N‐AHP and subsequently ranked using TOPSIS. To the best of our knowledge, no prior research has been undertaken on the utilization of Aronia fruit in such a study. The data acquired from this research will make a valuable contribution to the discipline of medicine as well as the food and pharmaceutical sectors.

## 2 Materials and Methods

### 2.1 Plant Material

Aronia fruit ( *Aronia melanocarpa* ) was obtained from a local market. The fresh Aronia fruits were subjected to freezing at a temperature of −18°C before the extraction process.

### 2.2 Reagents and Solvents

For the extraction and analysis of bioactive content, the following reagents and solvent were used: formic acid (98.0%–100%, Sigma‐Aldrich, St. Louis, MO, USA), ultrapure water (Milli‐Q, Merck Millipore, St. Louis, MO, USA), methanol (HPLC gradient grade, J.T. Baker, Landsmeer, Holland), ethanol (95%, Constanta Pharm M, Moscow, Russia), acetone (≥ 97%, Sigma‐Aldrich), Folin–Ciocalteu reagent (Sigma‐Aldrich), sodium carbonate anhydrous (chemically pure, Vekton, Russia), sodium nitrite (Sigma‐Aldrich), aluminum chloride (Sigma‐Aldrich), sodium hydroxide (Sigma‐Aldrich), DPPH (Sigma‐Aldrich), copper(II) chloride (Merck Millipore), ammonium acetate (Merck Millipore), neocuproine (2,9‐dimethyl‐1,10‐phenanthroline) (Sigma‐Aldrich). Reagents used for antimicrobial activity: Nutrient broth, Nutrient agar, Sabouraud Dextrose Agar, and Sabouraud Dextrose Broth were obtained from Merck. For in vitro cytotoxicity, Dulbecco's modified Eagle's medium (DMEM), fetal bovine serum (FBS), penicillin–streptomycin, trypsin/EDTA (0.25%), and Trypan Blue were acquired from Gibco Life Technologies (Gaithersburg, Maryland, USA) and 2,3‐bis‐(2‐methoxy‐4‐nitro‐5‐sulfophenyl)‐2H‐tetrazolium‐5‐carboxanilide (XTT) was from Biomatik.

### 2.3 Extraction Procedure of Aronia Fruit

Aronia fruits that had been frozen were meticulously crushed within a mortar until they reached a state of puree. Three distinct solvents were created for the purpose of extraction. The three extracts used in this study were the methanolic extract (ME) prepared with 80% methanol and 1% formic acid, the ethanolic extract (EE) prepared with 80% ethanol, and the acetone extract (AE) prepared with 70% acetone. Pureed samples (5 g) were incorporated with 50 mL of pre‐prepared solvents. The extraction procedure was conducted using a shaker operating at a speed of 150 rpm at ambient temperature for the duration of one night. Each extract underwent centrifugation at 14,000 *g* for a duration of 10 min. The supernatants were then passed through a 0.45‐μm filter to ensure clarity. A volume of 5 mL from the final produced extracts was set aside for further bioactivity investigations. The residual volume of 45 mL of the extract was subjected to drying using an oven at 30°C, following which aqueous extracts were then produced for cell culture and antimicrobial activity. The main aqueous stock concentration of ME of Aronia (MEA) was established at 0.479 g/mL, while the primary aqueous stock concentration of EE of Aronia (EEA) was set at 0.512 g/mL, and AE of Aronia (AEA) was formulated at 0.457 g/mL.

### 2.4 Determination of Total Phenolic Content Amount

To ascertain the total phenolic content (TPC) of Aronia fruit, a mixture was prepared by combining 0.5 mL of the fruit extract with 2.5 mL of Folin–Ciocalteu phenol reagent (0.2 N) and 2.0 mL of Na_2_CO_3_ (7.5%) in a tube. The resulting mixture was thereafter incubated in darkness for a duration of 30 min (Singleton et al. 1999). The absorbance of the extracts was measured at a wavelength of 760 nm using a Shimadzu UV‐1800 UV–Vis spectrophotometer, manufactured in Kyoto, Japan. The phenolic content was quantified in milligrams of gallic acid equivalent (GAE) per gram of sample (mg GAE/g).

### 2.5 Determination of Total Flavonoid Content Amount

The method provided by Zhishen et al. (1999) was used to evaluate the total flavonoid content (TFLC) of the samples. As per the prescribed procedure, 1 mL of the extract was combined with 4 mL of distilled water, followed by the addition of 0.3 mL of a 5% NaNO_2_ solution and 0.3 mL of a 10% AlCl_3_ solution. The resulting mixture was then allowed to stand at ambient temperature for a duration of 6 min. Following the addition of 2 mL of NaOH (1 M), the total volume was subsequently augmented to 10 mL through the addition of distilled water. The absorbance of extracts at a wavelength of 510 nm was measured using a spectrophotometer. The results are presented in milligrams of catechin equivalent (ce) per gram (mg ce/g).

### 2.6 Determination of Total Antioxidant Capacity

The determination of the antioxidant capacity of the extracts was conducted through the utilization of the 1,1‐diphenyl‐2‐picrylhydrazyl (DPPH) radical scavenging activity and the copper‐reducing antioxidant capacity (CUPRAC) tests. In the DPPH analysis, a volume of 0.1 mL of the extract was introduced to a solution containing 4.9 mL of DPPH dissolved in methanol at a concentration of 0.1 mmol/L. Subsequently, the mixture was incubated for a duration of 20 min under conditions of darkness (Singh et al. 2002). A UV–vis spectrophotometer was used to measure the absorbance of the extracts at 517 nm. Data are expressed as mg trolox equivalents (TE) per gram (mg TE/g).

In the CUPRAC study, a 0.1‐mL aliquot of the extract was introduced into a mixture containing 1 mL of CuCl_2_ (10 mmol/L), 1 mL of neocuproin (7.5 mmol/L), and 1 mL of NH_4_Ac (1 mol/L). The resulting solution was then brought to a total volume of 4.1 mL by adding 1 mL of water (Apak et al. 2004). Following a period of incubation in a light‐restricted environment for a duration of 1 h, the absorbance of the samples was quantified at a wavelength of 450 nm. The data are given in units of milligram trolox equivalents (TE) per gram (mg TE/g).

### 2.7 Determination of Total Anthocyanin Content

The quantification of anthocyanin content in the extracts was conducted by analyzing the absorbance values obtained at various pH levels (Wrolstad et al. 2005). Briefly, 1 mL of the extracted solution was transferred into a 10 mL volumetric flask to prepare two dilutions: one with potassium chloride buffer at pH 1.0 and the other with sodium acetate buffer at pH 4.5, diluting each as required. These dilutions were permitted to equilibrate for a duration of 15 min. The measurement of absorbance for the extracts was conducted at wavelengths of 520 and 700 nm.

The calculation of the absorbance of the diluted sample (*A*) was conducted as follows:

$$A={\left(A_{520}-A_{700}\right)}_{\mathrm{pH}\;1.0}-{\left(A_{520}-A_{700}\right)}_{\mathrm{pH}\;4.5}$$

The calculation of the total anthocyanin content (TAC) in the samples was performed using the equation shown below:

$$\text{Anthocyanin content}\;\left(\mathrm{mg}/L\right)=\frac{A\times \mathrm{MW}\times \mathrm{DF}\times 1000}{\varepsilon \times 1}$$

The results were expressed as cyanidin‐3‐glucoside equivalents for the extracts using the absorbance value (*A*), molecular weight (MW) of 449.2 g/mol, absorptive coefficient (*ε*) of 26,900 L/mol, and dilution factor (Allen et al. 2013) of the samples.

### 2.8 Determination of Antimicrobial Activity

The antimicrobial activity of Aronia extracts was assessed using the agar well diffusion method (Durak and Uçak 2015). All extracts were utilized for antimicrobial activity testing following adjustment to a concentration of 0.45 g/mL.

*Bacillus cereus* FMC19, *Staphylococcus aureus* ATCC 25923, *Listeria monocytogenes* ATCC 19118, *Salmonella typhimurium* ATCC 14028, *Escherichia coli* O157:H7 ATCC 33150, *Candida albicans* ATCC 10251, and *Saccharomyces cerevisiae* ATCC 9753 were employed to assess the antimicrobial activity of Aronia extracts. The activation of bacterial strains was achieved by subjecting them to two incubation cycles in Nutrient Broth at 37°C for 18 h. The activation of yeasts was achieved by subjecting them to a two‐step incubation process in Sabouraud Dextrose Broth at a temperature of 30°C for 24–48 h. The Nutrient Agar and Sabouraud Dextrose Agar were subjected to autoclaving and subsequently cooled to a temperature range of 43°C–45°C. Following this, the agar media were introduced with bacteria and yeast to achieve a final cell concentration of 10^6^–10^7^ colony‐forming units per milliliter (CFU/mL). Following the solidification of the agar, four wells were generated within it utilizing a sterilized cork borer with a diameter of 4 mm. The aqueous extracts (50 μL) were transferred into wells and thereafter subjected to incubation at a temperature of 37°C for a duration of 24 h in the case of bacteria, and at a temperature of 30°C for a duration ranging from 24 to 48 h for yeasts. The antimicrobial activity was assessed by measuring the inhibition zone (mm) against the test microorganism.

### 2.9 In Vitro Cell Culture

The Caco‐2 adenocarcinoma cell line was employed to assess the cytotoxic effects of the extracts. The Caco‐2 adenocarcinoma cell line was grown in DMEM supplemented with 10% (v/v) FBS and 0.5% (v/v) penicillin (10,000 units/mL) and streptomycin (10,000 μg/mL). The cells were cultivated in cell culture flasks with a surface area of 25 cm^2^ and incubated at 37°C with a CO_2_ concentration of 5% in an environment with a 95% humidity for a duration of 24 h. The cells, which had reached 80%–90% confluency, were subjected to treatment with a 0.25% Trypsin–EDTA (1×) solution to detach them from the surface of the flask. Subsequently, the cells were centrifuged at a speed of 1000 rpm for a duration of 5 min. The supernatant was removed, and the quantity of living cells in the pellet was determined using a hemocytometer with the trypan blue staining procedure.

### 2.10 Cell Viability Assay

The in vitro cytotoxicity of Aronia extracts was conducted using the XTT assay, incorporating several alterations to the previously documented experimental procedures (Cakir‐Koc et al. 2018). Caco‐2 cells were carefully transferred onto 96‐well flat‐bottom microplates at a density of 10^4^ cells per well using a pipette. To facilitate cellular adhesion to the wells, the cells were subjected to incubation at 37°C for 24 h. Following the completion of the incubation procedure, various concentrations of the samples in DMEM (10, 8, 6, 4, 2, 1, and 0.5 mg/mL) were introduced into individual wells. Subsequently, the samples were subjected to a further 24 h incubation period. Following the incubation period, a volume of 100 μL of medium was removed from the wells. Subsequently, 100 μL of an XTT solution with a concentration of 0.5 mg/mL, made using fresh medium, was introduced into each well. Following incubation of the microplates at 37°C for 3 h, the optical density (OD) was assessed at a wavelength of 450 nm utilizing a microplate reader (Elisa reader Elx800, Biotek, Vermont, USA). Subsequently, the calculation of the viable cell count was performed based on the application of the subsequent formula:

$$\text{Cell viability}\;\left(\%\right)=\left({\mathrm{OD}}_{\text{sample}}/{\mathrm{OD}}_{\text{control}}\right)\times 100$$

### 2.11 Statistical Analysis

The results are reported in the form of the mean value ± the standard deviation for each Aronia extract. The dissimilarities among the samples were assessed utilizing a one‐way analysis of variance (ANOVA) and Tukey's comparison test, with a significance level of 95%.

The outcomes derived from the extraction of Aronia fruit using three distinct solvents were evaluated in conjunction with the N‐AHP‐based TOPSIS method. The study conducted a comparison utilizing actual data. The parameters employed to identify the optimal response, along with their corresponding definitions, are presented in Table 1.

**TABLE 1:** Criteria codes and their corresponding descriptions.

Codes	Criteria name
*C*_1_	DPPH
*C*_2_	TPC
*C*_3_	CUPRAC
*C*_4_	TFLC
*C*_5_	TAC
*C*_6_	IC50
*C*_7_	*S. aureus*
*C*_8_	*B. cereus*
*C*_9_	*L. monocytogenes*

#### 2.11.1 Multi‐Criteria Decision‐Making Method (MCDM)

The term “multi‐criteria decision‐making” refers to a decision‐making procedure that involves taking into consideration several aspects of the situation. During this phase of the process, the person responsible for making decisions assesses several criteria, decides the significance of each of these factors, and then evaluates possibilities in line with these criteria.

The study aims to obtain a common evaluation by obtaining opinions from three experts in the field and expecting the evaluation of many criteria among themselves. In such studies, the AHP method, which is well accepted in the literature, has been preferred. Numerous research studies, including those by Slamaa et al. (2021), demonstrate that the neutrosophic AHP approach yields more precise and reliable outcomes compared to traditional AHP and FAHP procedures. Consequently, the neutrosophic AHP approach was used for the purpose of this research. The neutrosophic AHP approach is a methodology employed in multi‐criteria decision‐making procedures. The approach offers a rating and assessment utilizing neutrosophic weights and neutrosophic scores based on the criteria established by the decision‐maker. This approach facilitates a more nuanced and realistic decision‐making process by accounting for varying degrees of uncertainty and ambiguity (Slamaa et al. 2021).

##### 2.11.1.1 Neutrosophic Set Theory

Some basic definitions of NS theory are provided in this section (Vafadarnikjoo 2020).

**Definition 1**. Let *U* be a finite set of objects and let *x* signify a generic element in *U*. The NS *A* in *U* is characterized by a truth‐membership function *T*_*A*_(*x*), an in‐determinacy‐membership function *I*_*A*_(*x*), and a falsity‐membership function $F_{A}\left(x\right).T_{A}\left(x\right),I_{A}\left(x\right)$, and $F_{A}\left(x\right)$ are the elements of $]0^{-},1^{+}[$. It can be shown as (Smarandache 1999):

(1) $$A\;=\left\{\left\langle x,\left(T_{A}\left(x\right),I_{A}\left(x\right),F_{A}\left(x\right)\right)\right\rangle :x\;U,T_{A}\;\left(x\right),I_{A}\;\left(x\right),F_{A}\in ]0^{-},1^{+}\left[\;\right\},\right.$$

Note that $0^{-}\leq T_{A}\left(x\right)+I_{A}\left(x\right)+F_{A}\left(x\right)\leq 3^{+}$.

**Definition 2**. Let *U* be a finite set of elements and let *x* signify a generic element in *U*. A single‐valued neutrosophic set (SVNS) *A* in *U* is defined by a truth‐membership function $T_{A}\left(x\right)$, an indeterminacy‐membership function $I_{A}\left(x\right)$, and a falsity‐membership function $F_{A}\left(x\right).T_{A}\left(x\right),I_{A}\left(x\right)$, and $F_{A}\left(x\right)$ are the elements of $\left[0,1\right]$. It can be shown as (Wang et al. 2010):

(2) $$\begin{array}{rr} A & =\left\{\left\langle x,\left(T_{A}\left(x\right),I_{A}\left(x\right),F_{A}\left(x\right)\right)\right\rangle :x\;\epsilon \;U,T_{A}\;\left(x\right),I_{A}\;\left(x\right),F_{A}\left(x\right)\in \left[0,1\right]\right\} \end{array},$$

Note that $0\leq T_{A}\left(x\right)+I_{A}\left(x\right)+F_{A}\left(x\right)\leq 3$.

For convenience, an SVNS $A=\left\{\left\langle x,\left(T_{A}\left(x\right)\right.,\left.I_{A}\left(x\right),F_{A}\left(x\right)\right)\right\rangle :x\in U\right\}$ is sometimes shown as a $A=\left\{\left\langle T_{A}\left(x\right),I_{A}\left(x\right),F_{A}\left(x\right)\right\rangle :x\in U\right\}$ called the simplified form.

**Definition 3**. An SVTNN, $\begin{array}{c} \overset{`}{a}=\left\langle \left(a_{1},b_{1},c_{1},d_{1}\right);w_{\overset{`}{a}},u_{\tilde{a}},y_{\overset{`}{a}}\right\rangle , \end{array}$ $\begin{array}{c} a_{1},b_{1},c_{1},d_{1}\in R,a_{1}\leq b_{1}\leq c_{1}\leq d_{1} \end{array}$, and $w_{\overset{ˋ}{a}},u_{\overset{ˋ}{a}},y_{\overset{ˋ}{a}}\in \left[0,1\right]$ is a particular single‐valued neutrosophic number (SVNN) whose $T_{\overset{ˋ}{a}}\left(x\right)$, $I_{\overset{ˋ}{a}}\left(x\right)$, and $F_{\overset{ˋ}{a}}\left(x\right)$ are presented as the following equations, respectively (Deli and Subas 2014):

(3) $$T_{\overset{ˋ}{a}}\left(x\right)=\left\{\begin{array}{ll} \frac{\left(x-a_{1}\right)w_{\overset{ˋ}{a}}}{\left(b_{1}-a_{1}\right)}, & a_{1}\leq x<b_{1},\\ w_{\overset{ˋ}{a}}, & b_{1}\leq x\leq c_{1},\\ \frac{\left(d_{1}-x\right)w_{\overset{ˋ}{a}}}{\left(d_{1}-c_{1}\right)}, & c_{1}<x\leq d_{1},\\ 0, & \\ 0, & \end{array}\right.$$

(4) $$I_{\overset{ˋ}{a}}\left(x\right)=\left\{\begin{array}{ll} \frac{\left(b_{1}-x+u_{\overset{ˋ}{a}}\left(x-a_{1}\right)\right)}{\left(b_{1}-a_{1}\right)}, & a_{1}\leq x<b_{1},\\ u_{\overset{ˋ}{a}}, & b_{1}\leq x\leq c_{1},\\ \frac{\left(x-c_{1}+u_{\overset{ˋ}{a}}\left(d_{1}-x\right)\right)}{\left(d_{1}-c_{1}\right)}, & c_{1}<x\leq d_{1},\\ 1, & \;\text{otherwise}, \end{array}\right.$$

(5) $$F_{\tilde{a}}\left(x\right)=\left\{\begin{array}{ll} \frac{\left(b_{1}-x+y_{\overset{ˋ}{a}}\left(x-a_{1}\right)\right)}{\left(b_{1}-a_{1}\right)}, & a_{1}\leq x<b_{1},\\ y_{\tilde{a}}, & b_{1}\leq x\leq c_{1},\\ \frac{\left(x-c_{1}+y_{\overset{ˋ}{a}}\left(d_{1}-x\right)\right)}{\left(d_{1}-c_{1}\right)}, & c_{1}<x\leq d_{1},\\ 1, & \;\text{otherwise}. \end{array}\right.$$

**Definition 4**. Given $\overset{ˋ}{a}=\left\langle \left(a_{1},b_{1},c_{1},d_{1}\right)w_{\overset{ˋ}{a}},u_{\overset{ˋ}{a}},y_{\overset{ˋ}{a}}\right\rangle$; and $\overset{ˋ}{b}=\left\langle \left(a_{2},b_{2},c_{2},d_{2}\right);w_{\overset{ˋ}{b}},u_{\tilde{b}},y_{\overset{ˋ}{b}}\right\rangle$, and $\begin{array}{c} \lambda >0,w_{\overset{`}{a}},w_{\overset{`}{a}},y_{a},w_{b},u_{\tilde{b}},y_{\overset{`}{b}} \end{array}$ $\begin{array}{c} \in \left[0,1\right],a_{1},b_{1},c_{1},d_{1},a_{2},b_{2},c_{2},d_{2}\in \mathbb{R},a_{1}\leq b_{1}\leq c_{1}\leq d_{1} \end{array}$, and $a_{2}\leq b_{2}$ $\leq c_{2}\leq d_{2}$, and then Equations (6) and (7) are true (Ye 2017a):

(6) $$\overset{ˋ}{a}+\tilde{b}=\left\langle \left(a_{1}+a_{2},b_{1}+b_{2},c_{1}+c_{2},d_{1}+d_{2}\right);w_{\overset{ˋ}{a}}+w_{\overset{ˋ}{b}}-w_{\overset{ˋ}{a}}w_{\overset{ˋ}{b}},u_{\overset{ˋ}{a}}u_{\overset{ˋ}{b}},y_{\overset{ˋ}{a}}y_{\overset{ˋ}{b}}\right\rangle ,$$

(7) $$\lambda \overset{ˋ}{a}=\left\langle \left(\lambda a_{1},\lambda b_{1},\lambda c_{1},\lambda d_{1}\right);1-{\left(1-w_{\overset{ˋ}{a}}\right)}^{\lambda },u_{\overset{ˋ}{a}}^{\lambda },y_{\overset{ˋ}{a}}^{\lambda }\right\rangle$$

When $a_{1},b_{1},c_{1},d_{1},a_{2},b_{2},c_{2},d_{2}>0$, then Equations (8) and (9) are true:

(8) $$\overset{ˋ}{a}\overset{ˋ}{b}=\left\langle \left(a_{1}a_{2},b_{1}b_{2},c_{1}c_{2},d_{1}d_{2}\right);w_{\overset{ˋ}{a}}w_{\overset{ˋ}{b}},u_{\overset{ˋ}{a}}+u_{\overset{ˋ}{b}}-u_{\overset{ˋ}{a}}{\overset{ˋ}{\tau }}_{\overset{ˋ}{b}},y_{\overset{ˋ}{a}}+y_{\overset{ˋ}{b}}-y_{\overset{ˋ}{a}}y_{\overset{ˋ}{b}}\right\rangle$$

(9) $${\overset{ˋ}{a}}^{\lambda }=\left\langle \left(a_{1}^{\lambda },b_{1}^{\lambda },c_{1}^{\lambda },d_{1}^{\lambda }\right);w_{\overset{ˋ}{a}}^{\lambda },1-{\left(1-u_{\overset{ˋ}{a}}\right)}^{\lambda },1-{\left(1-y_{\overset{ˋ}{a}}\right)}^{\lambda }\right\rangle .$$

**Definition 5**. Given $\overset{ˋ}{a}=\left\langle \left(a,b,c,d\right);w_{a}^{\sim },u_{a}^{\sim },y_{a}^{\sim }\right\rangle$ and a,b,c,d>0. Then, the score function of $\overset{ˋ}{a}$ can be calculated in accordance with the following equation (Ye 2017a):

(10) $$S\left(\tilde{a}\right)=\frac{1}{12}\left(a+b+c+d\right)\left(2+w_{\overset{ˋ}{a}}-w_{\overset{ˋ}{a}}-y_{\overset{ˋ}{a}}\right),S\left(\tilde{a}\right)\in \left[0,1\right].$$

**Definition 6**. In order to compare two SVTNNs $\tilde{a}=\left\langle \left(a_{1},b_{1},c_{1},d_{1}\right);w_{\tilde{a}},u_{\tilde{a}},y_{\overset{ˋ}{a}}\right\rangle$, and $\tilde{b}=\left\langle \left(a_{2},b_{2},c_{2},d_{2}\right);w_{\tilde{b}},u_{\overset{ˋ}{b}},y_{\overset{ˋ}{b}}\right\rangle$ where $a_{1},b_{1},c_{1},d_{1},a_{2},b_{2},c_{2},d_{2}>0$, then according to Equation (10), the score functions will be computed, and if $S\left(\tilde{a}\right)>S\left(\tilde{b}\right)$, then $\tilde{a}>\tilde{b}$; if $S\left(\tilde{a}\right)=S\left(\tilde{b}\right)$, then $\tilde{a}=\vec{b}$ (Ye 2017a).

**Definition 7**. Let ${\overset{ˋ}{a}}_{j}=\left\langle \left(a_{j},b_{j},c_{j},d_{j}\right);w_{{\overset{ˋ}{a}}_{i}},u_{{\overset{ˋ}{a}}_{j}},y_{{\tilde{a}}_{j}}\right\rangle \left(j=1,2,\ldots ,n\right)$ be a set of SVTNNs, then a trapezoidal neutrosophic weighted arithmetic averaging (TNWAA) operator is computed on the basis of (Ye 2017a):

(11) $$\text{TNWAA}\left({\overset{ˋ}{a}}_{1},{\overset{ˋ}{a}}_{2},\ldots ,{\tilde{a}}_{n}\right)=\sum_{j=1}^{n}\;\;p_{j}{\tilde{a}}_{j}=\left\langle \left(\sum_{j=1}^{n}p_{j}b_{j},\sum_{j=1}^{n}p_{j}c_{j},\sum_{j=1}^{n}p_{j}a_{j},\sum_{j=1}^{n}p_{j}d_{j}\right);1-\prod_{j=1}^{n}\;\;{\left(1-w_{a_{j}}\right)}^{p_{j}},\prod_{j=1}^{n}\;\;u_{a_{j}}^{p_{j}},\prod_{j=1}^{n}\;y_{{\overset{ˋ}{a}}_{j}}^{p_{j}}\right\rangle$$

where $p_{j}$ is the weight of ${\tilde{a}}_{j}\left(j=1,2,\ldots ,n\right)$ while $p_{j}>0$, and $\sum_{j=1}^{n}\;p_{j}=1$.

##### 2.11.1.2 Subtraction, Division, and Inverse of SVTNNs

The subtraction and division of simplified SVNNs (or single‐valued neutrosophic values) and SVNSs are introduced by Smarandache (2016) and (Ye (2017b)), respectively. Rani and Garg (2019) also studied subtraction and division operations on interval neutrosophic sets. In this section, subtraction, division, and the inverse of SVTNNs in general non‐simplified form are defined.

###### 2.11.1.2.1 Subtraction of SVTNNs

Let $\overset{`}{a}=\left\langle \left(a_{1},b_{1}\right.\right.,\left.c_{1},d_{1}\right);$ $w_{\overset{`}{a}},w_{\overset{`}{a}},y_{\overset{`}{a}}\rangle$, and $\overset{ˋ}{b}=\left\langle \left(a_{2},b_{2},c_{2},d_{2}\right);w_{\overset{ˋ}{b}},u_{\overset{ˋ}{b}},y_{\overset{ˋ}{b}}\right\rangle$ be two SVTNNs, and $w_{a},w_{\overset{ˋ}{a}},y_{\tilde{a}},w_{-},\psi_{\overset{ˋ}{b}},y_{b}\in \left[0,1\right]$ with the restrictions that $w_{-}\neq 1,v_{b}\neq 0,y_{b}\neq 0$, and 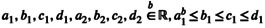, and $a_{2}\leq b_{2}\leq c_{2}\leq d_{2}$, then the subtraction of the two SVTNNs is shown in Vafadarnikjoo and Scherz (2021):

(12) $$\overset{ˋ}{a}-\tilde{b}=\left\langle \left(a_{1}-d_{2},b_{1}-c_{2},c_{1}-b_{2},d_{1}-a_{2}\right);\frac{w_{\overset{ˋ}{a}}-w_{\overset{ˋ}{b}}}{1-w_{\overset{ˋ}{b}}},\frac{w_{\overset{ˋ}{a}}}{w_{\overset{ˋ}{b}}^{*}},\frac{y_{\overset{ˋ}{a}}}{y_{\overset{ˋ}{b}}^{∼}}\right\rangle$$

Note: for a negative value, replace it with zero. For a value of over one, replace it with one.

Proof. Let us consider Equation (13) where

(13) $$\begin{array}{rr} & \overset{ˋ}{a}=\left\langle \left(a_{1},b_{1},c_{1},d_{1}\right);w_{\overset{ˋ}{a}},w_{\tilde{a}},y_{\overset{ˋ}{a}}\right\rangle ;\overset{ˋ}{b}=\left\langle \left(a_{2},b_{2},c_{2},d_{2}\right);w_{\overset{ˋ}{b}},w_{\tilde{b}},y_{\overset{ˋ}{b}}\right\rangle ;\\ & \tilde{c}=\left\langle \left(x,r,z,s\right);w_{\tilde{c}},w_{\tilde{c}},y_{\tilde{c}}\right\rangle \\ & \tilde{a}-\tilde{b}=\tilde{c}. \end{array}$$

By adding neutrosophically, $\tilde{b}$ to the sides of Equations ((13), (14), (15), (16)) results,

(14) $$\overset{ˋ}{a}=\overset{ˋ}{c}+\overset{ˋ}{b}=\left\langle \left(x+a_{2},r+b_{2},z+c_{2},s+d_{2}\right);w_{\overset{ˋ}{c}}+w_{\overset{ˋ}{b}}-w_{\overset{ˋ}{c}}w_{\overset{ˋ}{b}},u_{\tau }u_{\overset{ˋ}{b}},y_{\overset{ˋ}{c}}y_{\overset{ˋ}{b}}\right\rangle .$$

Then,

(15) $$\tilde{a}=\left\langle \left(a_{1},b_{1},c_{1},d_{1}\right);w_{a},u_{\tilde{a}},y_{\tilde{a}}\right\rangle =\left\langle \left(x+a_{2},r+b_{2},z+c_{2},s+d_{2}\right);w_{\overset{ˋ}{c}}+w_{\overset{ˋ}{b}}-w_{\tilde{c}}w_{\tilde{b}},u_{c}\tau_{-},y_{\tilde{c}}y_{\tilde{b}}\right\rangle$$

and

(16) $$\begin{array}{c} \left\{\begin{array}{l} w_{\overset{ˋ}{a}}=w_{\overset{ˋ}{c}}+w_{\overset{ˋ}{b}}-w_{\overset{ˋ}{c}}w_{\overset{ˋ}{b}}\Rightarrow w_{\overset{ˋ}{a}}-w_{\overset{ˋ}{b}}=w_{\overset{ˋ}{c}}\left(1-w_{\overset{ˋ}{b}}\right)\Rightarrow w_{\overset{ˋ}{c}}=\frac{w_{\overset{ˋ}{a}}-w_{\overset{ˋ}{b}}}{1-w_{\overset{ˋ}{b}}},\\ u_{\overset{ˋ}{a}}=u_{c}{\overset{ˋ}{u}}_{b}\Rightarrow u_{c}=\frac{u_{\overset{ˋ}{a}}}{u_{\overset{ˋ}{b}}},\\ y_{\overset{ˋ}{a}}=y_{c}y_{b}\Rightarrow y_{c}=\frac{y_{\overset{ˋ}{a}}}{y_{\overset{ˋ}{b}}} \end{array}\right. \end{array}$$

It is concluded that $\begin{array}{c} -\overset{`}{b}=\left\langle \left(-d_{2},-c_{2},--a_{2}\right)\right.;\left(w_{b}/w_{b}-1\right), \end{array}$ $\begin{array}{c} \left(1/u_{b}\right),\left.\left(1/y_{b}^{-}\right)\right\rangle \end{array}$, noting the remark above.

###### 2.11.1.2.2 Division of SVTNNs

Let $\overset{`}{a}=\left\langle \left(a_{1},b_{1},c_{1},d_{1}\right)\right.;$ $w_{\overset{`}{a}},u_{\overset{`}{a}},\left.y_{\overset{`}{a}}\right\rangle$ and $\tilde{b}=\left\langle \left(a_{2},b_{2},c_{2},d_{2}\right);w_{\overset{ˋ}{b}},u_{\overset{ˋ}{b}},y_{\overset{ˋ}{b}}\right\rangle$ be two SVTNNs where $a_{1},b_{1},c_{1},d_{1},a_{2},b_{2},c_{2},d_{2}>0$, $a_{1}\leq b_{1}\leq c_{1}\leq d_{1},$ $a_{2}\leq b_{2}\leq c_{2}\leq d_{2}$, and $w_{\overset{ˋ}{a}},u_{\tilde{a}},y_{\tilde{a}},w_{\tilde{b}},u_{b},y_{b}\in \left[0,1\right]$ with the restrictions that $w_{b}\neq 1,v_{b}\neq 0$, and $y_{b}\neq 0$, then the division of the two SVTNNs is shown in

(17) $$\overset{ˋ}{a}÷\tilde{b}=\left\langle \left(\frac{a_{1}}{d_{2}},\frac{b_{1}}{c_{2}},\frac{c_{1}}{b_{2}},\frac{d_{1}}{a_{2}}\right);\frac{w_{\overset{ˋ}{a}}}{w_{\overset{ˋ}{b}}},\frac{u_{\overset{ˋ}{a}-}\psi_{\overset{ˋ}{b}}}{1-u_{\overset{ˋ}{b}}},\frac{y_{\overset{ˋ}{a}}-y_{\overset{ˋ}{b}}}{1-y_{\overset{ˋ}{b}}}\right\rangle$$

Note: for a negative value, replace it with zero. For a value of over one, replace it with one.

Proof. Let us consider Equation (18) where

(18) $$\begin{array}{c} \tilde{a}=\left\langle \left(a_{1},b_{1},c_{1},d_{1}\right);w_{\tilde{a}},u_{\tilde{a}},y_{\tilde{a}}\right\rangle ;\tilde{b}=\left\langle \left(a_{2},b_{2},c_{2},d_{2}\right);w_{\tilde{b}},u_{\tilde{b}},y_{\tilde{b}}\right\rangle \\ \tilde{c}=\left\langle \left(x,r,z,s\right);w_{\tilde{c}},w_{\tilde{c}},y_{\tilde{c}}\right\rangle \\ \tilde{a}÷\tilde{b}=\tilde{c} \end{array}$$

By multiplying neutrosophically, $\tilde{b}$ to the sides of Equations ((18), (19), (20), (21)) is obtained:

(19) $$\overset{ˋ}{a}=\overset{ˋ}{c}\times \overset{ˋ}{b}=\left\langle \;\left(xa_{2},rb_{2},zc_{2},sd_{2}\right);w_{\overset{ˋ}{c}}w_{\overset{ˋ}{b}},u_{\overset{ˋ}{c}}+u_{\overset{ˋ}{b}}-u_{\overset{ˋ}{c}}u_{\overset{ˋ}{b}},y_{\overset{ˋ}{c}}+y_{\overset{ˋ}{b}}-y_{c}y_{\overset{ˋ}{b}}\right\rangle$$

Then,

(20) $$\overset{ˋ}{a}=\left\langle \left(a_{1},b_{1},c_{1},d_{1}\right);w_{\overset{ˋ}{a}},u_{\overset{ˋ}{a}},y_{\overset{ˋ}{a}}\right\rangle =\left\langle \left(xa_{2},rb_{2},zc_{2},sd_{2}\right);w_{\overset{ˋ}{c}}w_{\overset{ˋ}{b}},u_{\overset{ˋ}{c}}+u_{\overset{ˋ}{b}}-u_{\overset{ˋ}{c}}u_{\overset{ˋ}{b}},y_{\overset{ˋ}{c}}+y_{\overset{ˋ}{b}}-y_{c}y_{\overset{ˋ}{b}}\right\rangle$$

and

(21) $$\left\{\begin{array}{c} w_{\overset{ˋ}{a}}=w_{\overset{ˋ}{c}}w_{\overset{ˋ}{b}}\Rightarrow w_{\overset{ˋ}{c}}=\frac{w_{\overset{ˋ}{a}}}{w_{\overset{ˋ}{b}}},\\ u_{\overset{ˋ}{a}}=u_{\overset{ˋ}{c}}+u_{\overset{ˋ}{b}}-u_{\overset{ˋ}{c}}u_{\overset{ˋ}{b}}\Rightarrow u_{\overset{ˋ}{a}}-u_{\overset{ˋ}{b}}=u_{\overset{ˋ}{c}}\left(1-u_{\overset{ˋ}{b}}\right)\Rightarrow u_{\overset{ˋ}{c}}=\frac{u_{\overset{ˋ}{a}}-u_{\overset{ˋ}{b}}}{1-u_{\overset{ˋ}{b}}}\\ y_{\overset{ˋ}{a}}=y_{\overset{ˋ}{c}}+y_{\overset{ˋ}{b}}-y_{\overset{ˋ}{c}}y_{\overset{ˋ}{b}}\Rightarrow y_{\overset{ˋ}{a}}-y_{\overset{ˋ}{b}}=y_{\overset{ˋ}{c}}\left(1-y_{\overset{ˋ}{b}}\right)\Rightarrow y_{\overset{ˋ}{c}}=\frac{y_{\overset{ˋ}{a}}-y_{\overset{ˋ}{b}}}{1-y_{\overset{ˋ}{b}}}. \end{array}\right.,$$

###### 2.11.1.2.3 Inverse of an SVTNN

Let $\overset{`}{a}=\left\langle \left(a_{1},b_{1}\right.\right.,\left.c_{1},d_{1}\right);$ $w_{\overset{`}{a}},w_{\overset{`}{a}},y_{\overset{`}{a}}\rangle$, be an SVTNN where $a_{1},b_{1},c_{1},d_{1}>0$, $a_{1}\leq b_{1}\leq c_{1}\leq d_{1}$, and $w_{\overset{ˋ}{a}},u_{\overset{ˋ}{a}},y_{\overset{ˋ}{a}}\in \left[0,1\right]$, then the inverse of $\overset{ˋ}{a}$ is represented in

(22) $${\overset{ˋ}{a}}^{-1}=\frac{1}{\overset{ˋ}{a}}=\left\langle \left(\frac{1}{d_{1}},\frac{1}{c_{1}},\frac{1}{b_{1}},\frac{1}{a_{1}}\right);\frac{1}{w_{\overset{ˋ}{a}}},\frac{u_{\overset{ˋ}{a}}}{u_{\overset{ˋ}{a}}-1},\frac{y_{\overset{ˋ}{a}}}{y_{a}-1}\right\rangle$$

Note: for a negative value, replace it with zero. For a value of over one, replace it with one.

Proof. Let us consider Equation (23), where $\overset{`}{a}=\left\langle \left(a_{1},b_{1},c_{1},d_{1}\right)\right.;$ $w_{\overset{`}{a}},u_{\overset{`}{a}},y_{\overset{`}{a}}\rangle$, and ${\overset{ˋ}{a}}^{-1}=\left\langle \left(x,r,z,s\right);w_{\tau },u_{\tau },y_{c}\;\right\rangle$,

(23) $${\overset{ˋ}{a}}^{-1}=\frac{1}{\tilde{a}}=\frac{\left\langle \left(1,1,1,1\right);1,0,0\right\rangle }{\left\langle \left(a_{1},b_{1},c_{1},d_{1}\right);w_{\overset{ˋ}{a}},w_{\overset{ˋ}{a}},y_{\overset{ˋ}{a}}\right\rangle }.$$

Then, based on the division rule of two SVTNNs referring to Equation (17), the proof is provided.

#### 2.11.2 Neutrosophic‐Analytical Hierarchy Process (N‐AHP)

Introduced by Smarandache (1999), neutrosophic sets integrate fuzzy sets and intuitionistic fuzzy sets. This approach is extensively employed in practical applications to manage partial, ambiguous, and inconsistent information (Broumi et al. 2018; Karadayi‐Usta 2022). Neutrosophic sets are distinguished as a successful approach, particularly in addressing complicated situations. This methodology was devised to address the constraints of conventional fuzzy logic systems and offer a more adaptable analytical framework. Consequently, it provides a substantial benefit in assessing data sets characterized by ambiguity and inconsistency.

Neutrosophic clusters enable specialists to articulate their ideas with greater precision by including the element of uncertainty. The level of ambiguity signifies the degree of discord among decision‐makers. The suggested methodology seeks to rectify anomalies in expert judgments by amalgamating the perspectives of several decision‐makers into a unified view, hence enhancing consistency. Simultaneously, in neutrosophic clusters, resources operate independently and do not engage with one another depending on their reactions (Smarandache 2021; Smarandache and Pramanik 2016).

The conventional AHP technique considers the definitive judgments of decision‐makers, but neutrosophic sets render these judgments more adaptable. This study utilizes neutrosophic sets to manage ambiguity, enhancing the flexibility of decision‐makers' judgments. A matrix is constructed for each criterion based on the relative significance of the criteria. The significance of one element in relation to another is articulated as the superior element. Neutrosophic AHP employs neutrosophic numbers to articulate the extent of uncertainty, facilitating a more accurate representation of uncertain scenarios (Saaty 1987). It is articulated with Saaty's 9‐point scale. Table 2 (Vafadarnikjoo et al. 2023) presents several linguistic characteristics utilized by decision‐makers together with the corresponding relevance weights derived from neutrosophic sets (Radwan et al. 2016).

**TABLE 2:** N‐AHP SVTNNs rating scale.

Numerical scale	Verbal scale	SVTNNs	Score function
1/9	Extremely less important	< (0.11,0.11,0.11,0.11); 1.0.0 >	0.11
1/8	Very very strongly less important	< (0.11,0.11,0.13,0.14); 1.0.0 >	0.12
1/7	Very strongly plus less important	< (0.11,0.13,0.14,0.17); 1.0.0 >	0.14
1/6	Strongly plus less important	< (0.13,0.14,0.17,0.2); 1.0.0 >	0.16
1/5	Strongly less important	< (0.14,0.17,0.2,0.25); 1.0.0 >	0.19
1/4	Moderately plus less important	< (0.17,0.20,0.25,0.33); 1.0.0 >	0.24
1/3	Moderately less important	< (0.14,0.17,0.33,0.50); 1.0.0 >	0.29
1/2	Weakly less important	< (0.20,0.25,0.5,1); 1.0.0 >	0.49
1	Equally important	< (1,1,1,1); 0.5,0.5,0.5 >	0.5
2	Weakly more important	< (1,2,4,5); 0.4,0.65,0.6 >	1.15
3	Moderately more important	< (2,3,6,7); 0.3,0.75,0.7 >	1.28
4	Moderately plus more important	< (3,4,5,6); 0.6,0.35,0.4 >	2.78
5	Strongly more important	< (4,5,6,7); 0.8,0.15,0.2 >	4.49
6	Strongly plus more important	< (5,6,7); 0.7,0.25,0.3 >	4.66
7	Very strongly plus more important	< (6,7,8); 0.9,0.1,0.1 >	6.75
8	Very very strongly more important	< (7,8,9); 0.85,0.1,0.15 >	7.15
9	Extremely more important	< (9,9,9); 1,0,0 >	9

**FIGURE 1:**
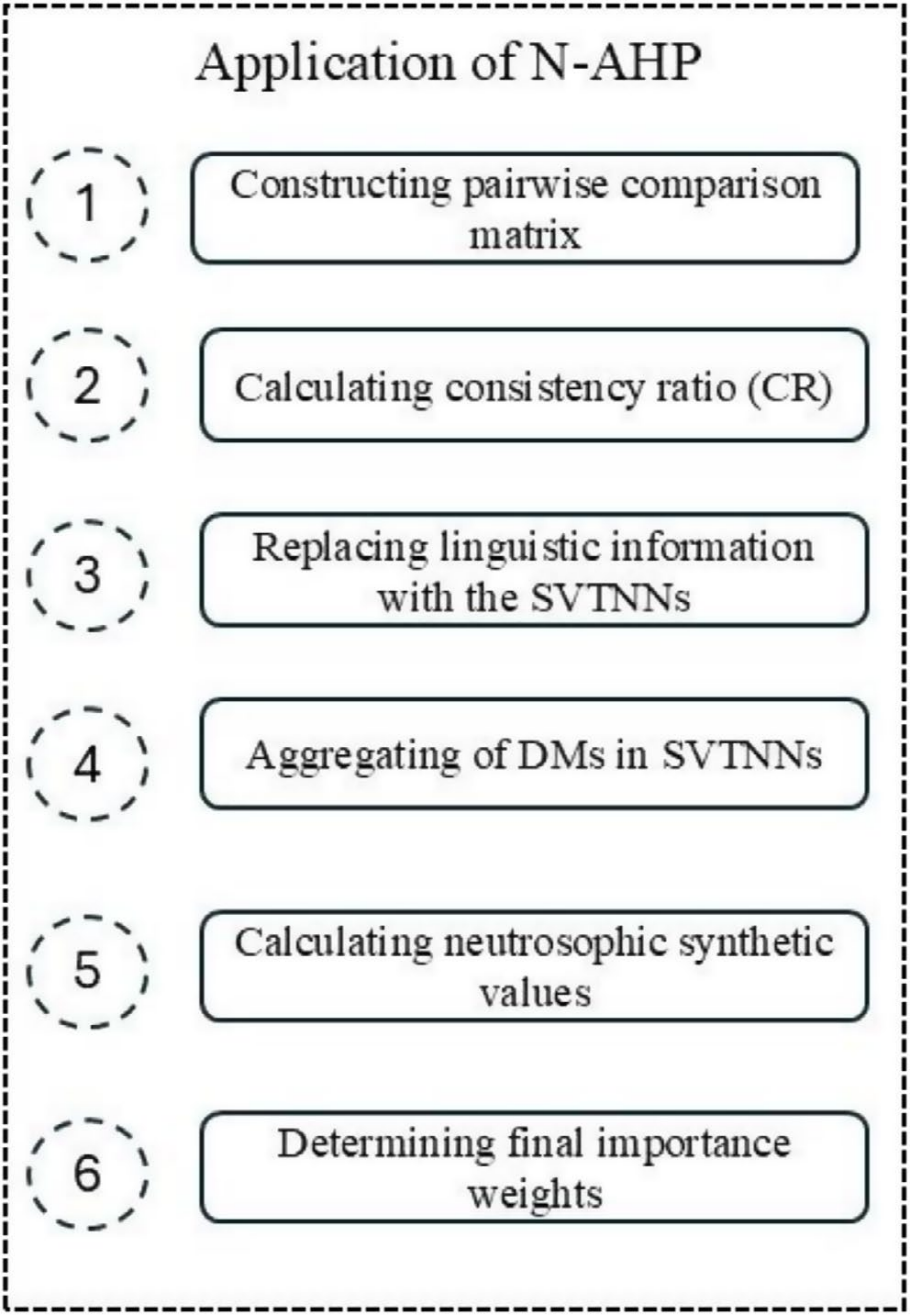
The procedural stages of the N‐AHP.

The N‐AHP method was carried out by applying the process step identified in Figure 1 (Vafadarnikjoo and Scherz 2021).

**Step 1**: Constructing a pairwise comparison matrix

In this phase, decision‐makers assess the components (i.e., alternatives or criteria) with the Saaty rating scale (Table 2). In expert judgment surveys, decision‐makers select a verbal term that indicates the relative importance of each factor in comparison to others. This procedure entails comparing assessments of the components.

For instance, criteria *C*_1_, *C*_2_, …, *C*_*n*_ and *a*_ijk_, as well as elements *a*_ijk_, *C*_*i*_ ve *C*_*j*_, are quantitatively assessed by the *k*th decision‐maker (*k* = 1, 2, …, *p*). This yields a bidirectional comparison matrix, as delineated in Equation (1) (Hayaty et al. 2014). In the expert judgment survey, decision‐makers select a language term that signifies the relative importance of each aspect in comparison to the others. A pairwise comparison matrix for the primary and secondary criteria is constructed utilizing the verbal scale and the associated neutrosophic numbers based on the perspectives of the decision‐makers. When several decision‐makers are involved in problem solving, the comparison matrices are consolidated into a unified common decision by geometric averaging or other recognized methodologies in the literature

(24) $$A_{k}=\left[a_{\mathrm{ijk}}\right]=\left[\begin{array}{cccc} 1 & a_{12k} & \cdots & a_{1\mathrm{nk}}\\ 1/a_{12k} & 1 & \ldots & a_{2\mathrm{nk}}\\ \vdots & \vdots & \ddots & \vdots \\ 1/a_{1\mathrm{nk}} & 1/a_{2\mathrm{nk}} & \cdots & 1 \end{array}\right]$$

**Step 2**: Determine the consistency rate (CR)

Considering the research carried out by Liberatore (1982), it is recommended that a consistency test be carried out to differentiate between consistent comparisons and inconsistent comparisons. This examination is depicted in Equation (25) as well as Table 3 (Golden et al. 1989). Decision‐makers are required to alter their judgments if the *CR* value is more than or equal to 0.1 (Leung and Cao 2000)

(25) $$\mathrm{CR}=\frac{\left(\left(\lambda_{\max}-n\right)/\left(n-1\right)\right)}{\mathrm{RI}}$$

**TABLE 3:** Random index table (*RI*).

*n*	1	2	3	4	5	6	7	8	9	10
*RI*	0	0	0.58	0.9	1.12	1.24	1.32	1.41	1.45	1.49

**Step 3**: Substituting verbal phrases with single‐valued trapezoidal neutrosophic numbers

The elements of the pairwise comparison matrices are substituted with the equivalent single‐valued trapezoidal neutrosophic numbers according to the scale presented in Table 2.

**Step 4**: Aggregating opinions of decision‐makers into single‐valued trapezoidal neutrosophic numbers

As seen in Equation (26), the trapezoidal neutrosophic weighted arithmetic mean operator is employed to integrate the viewpoints of decision‐makers (Ye 2017a)

(26) $$\begin{array}{c} \text{TNWAA}\;\left({\tilde{a}}_{1},{\tilde{a}}_{2},\ldots ,{\tilde{a}}_{n}\right)=\sum_{j=1}^{n}p_{j}{\tilde{a}}_{j}\\ =\left\langle \left(\sum_{j=1}^{n}p_{j}a_{j},\sum_{j=1}^{n}p_{j}b_{j},\sum_{j=1}^{n}p_{j}c_{j},\sum_{j=1}^{n}p_{j}d_{j}\right)\right.;\\ \left.1-\prod_{j=1}^{n}{\left(1-w_{{\tilde{a}}_{j}}\right)}^{p_{j}},\prod_{j=1}^{n}{\left(u_{{\tilde{a}}_{j}}\right)}^{p_{j}},\prod_{j=1}^{n}{\left(y_{{\tilde{a}}_{j}}\right)}^{p_{j}}\right\rangle \end{array}$$

**Step 5**: Neutrosophic synthetic values

The neutrosophic synthetic value of each element (*S*_*i*_) is computed using Equation (27) shown below. In the equation, *n* represents the quantity of elements, and *n*_*ij*_ denotes the (*i*, *j*)th member in the aggregated pairwise comparison matrix

(27) $$S_{i}=\sum_{j=1}^{n}n_{\mathrm{ij}}\times {\left[\sum_{i=1}^{n}\sum_{j=1}^{n}n_{\mathrm{ij}}\right]}^{-1},i=1,\ldots ,n$$

**Step 6**: Determine final significance weights

The ultimate significance weights are computed using Equation (28) and are represented as single‐valued trapezoidal neutrosophic numbers *W*_*i*_. Equation (29) is employed to compare the weights (Ye 2017a)

(28) $$W_{i}=\frac{S_{i}}{\sum_{i=1}^{n}S_{i}},i=1,\ldots ,n,$$

(29) $$S\;\left(\tilde{a}\right)=\frac{1}{12}\left(a+b+c+d\right)\;\left(2+w_{\tilde{a}}+u_{\tilde{a}}+y_{\tilde{a}}\right),S\;\left(\tilde{a}\right)\in \left[0,1\right]$$

#### 2.11.3 TOPSIS (Technique for Order Preference by Similarity to Ideal Solution)

In the TOPSIS analysis, three extracts were compared according to nine indicators. The values obtained in the N‐AHP analysis were used as a weight in the TOPSIS analysis. In N‐AHP, the dimension or indicator with the highest priority value is considered the indicator with the highest weight. All these weights are shown in Table 7. Before the TOPSIS analysis, a normalization process was applied to evaluate indicators with different units together. Thus, the values of each indicator are converted to be between 0 and 1 (Erdin and Ozkaya 2020a, 2020b). This methodology also prevents the analysis from being influenced by outliers. The process of normalization is shown in Formula (30). Afterwards, the data were analyzed in R Studio and MATLAB after editing in Excel

(30) $$X_{\text{normalized}}=\frac{\left(X-X_{\text{minimum}}\right)}{\left(X_{\text{maximum}}-X_{\text{minimum}}\right)}$$

TOPSIS (Technique for Order Preference by Similarity to Ideal Solution) was developed by Yoon and Hwang (1980). It involves a five‐step solution process. The steps of the TOPSIS method are described below (Erdin and Ozkaya 2017; Yoon 1980).

**Step 1**: Normalization of the decision matrix

The R matrix is built by employing the *r*_*ij*_ values computed in this stage:

(31) $$r_{\mathrm{ij}}=\frac{x_{\mathrm{ij}}}{\sqrt{\sum_{k=1}^{m}x_{\mathrm{kj}}^{2}}},i=1,\ldots ,m;j=1,\ldots ,n$$

**Step 2**: Computing *v*_*ij*_ matrix using the *v*_*ij*_‐weighted normal values. *w*_*j*_ defines the weight of the *j*th criterion or indicator.

(32) $$v_{\mathrm{ij}}=w_{j}r_{\mathrm{ij}},\sum_{j=1}^{n}w_{j}=1$$

**Step 3**: Creating ideal (A*) and negative ideal (*A* ^−^) solutions:

Finding the ideal solution set is shown in the following Formula (33):

(33) $$A^{*}=\left\{\left(\max_{i}v_{\mathrm{ij}}|j\in C_{b}\right),\left(\min_{i}v_{\mathrm{ij}}|j\in C_{c}\right)\right\}=\left\{\left.v_{j}^{*}\right|\;j=1,2,\ldots ,m\right\}$$

The set of negative ideal solutions is formed by selecting the smallest of the weighted evaluation factors in the V matrix. Creating the negative ideal solution set is shown in the following Formula (34):

(34) $$A^{-}=\left\{\left(\min_{i}v_{\mathrm{ij}}|j\in C_{b}\right),\left(\max_{i}v_{\mathrm{ij}}|j\in C_{c}\right)\right\}=\left\{\left.v_{j}^{-}\right|\;j=1,2,\ldots ,m\right\}$$

In both formulas, *J* represents the benefit (maximization) and *J*′ indicates the loss (minimization).

**Step 4:** In the TOPSIS method, Euclidian Distance Approach is used to find the deviations of the evaluation factor value for each decision point from the ideal and negative ideal solution set. The calculation of the ideal discrimination ($S_{i}^{*}$) measure is shown in Formula (35) and the calculation of the negative ideal discrimination ($S_{i}^{-}$) measure is shown in Formula (36):

(35) $$S_{i}^{*}=\sqrt{\sum_{j=1}^{m}{\left(v_{\mathrm{ij}}-v_{j}^{*}\right)}^{2},j=1,2,\ldots ,m}$$

(36) $$S_{i}^{-}=\sqrt{\sum_{j=1}^{m}{\left(v_{\mathrm{ij}}-v_{j}^{-}\right)}^{2},j=1,2,\ldots ,m}$$

**Step 5**: Determination of relative proximities to the A*. Then, the relative closeness (*RC*_*i*_) of the alternative defined as *A*_*i*_ to the ideal solution is obtained. Then, these values are ordered from largest to smallest.

(37) $${\mathrm{RC}}_{i}^{*}=\frac{S_{i}^{-}}{S_{i}^{*}+S_{i}^{-}},i=1,\ldots ,m$$

The value $C_{i}^{*}$ is in the range $0\leq C_{i}^{*}\leq 1$ and $C_{i}^{*}=1$ indicates the absolute proximity of the corresponding decision point to the ideal solution, and $C_{i}^{*}=0$ indicates the absolute proximity of the corresponding decision point to the negative ideal solution.

## 3 Results

The present research was conducted in two phases. Initially, Aronia fruit was extracted using three distinct solvents. The bioactive, antimicrobial, and cytotoxic characteristics were then assessed for each extract. The criteria for identifying the most appropriate solvent for Aronia fruit were assessed by professionals with the N‐AHP SVTNNs Rating Scale, followed by scoring and weighting. Subsequently, the ranking was conducted with TOPSIS based on the derived weightings.

Tables of data obtained from the first part of the study are given in the Supporting Information. Table S1 presents the variations in the amount of bioactive content of Aronia fruits when subjected to extraction using various solvents. The TPC amount of the EEA, MEA, and AEA samples was measured to be 721.74 ± 2.92, 934.03 ± 30.86, and 956.70 ± 11.65 mg GAE/g, respectively. The total antioxidant capabilities of Aronia fruits extracted with various solvents were determined using the DPPH and CUPRAC methods. The DPPH method was employed to determine the antioxidant capabilities of the EEA, MEA, and AEA samples, yielding values of 4816.13 ± 18.23, 5907.78 ± 10.16, and 5790.91 ± 50.37 mg TE/g, respectively. In accordance with the CUPRAC method, the total antioxidant capacity of EEA, MEA, and AEA was determined to be 2486.99 ± 39.08, 2582.25 ± 20.93, and 3554.07 ± 18.72 mg TE/g, respectively. The effect of different solvents on the total flavonoid amount (TFLC) and TAC of Aronia fruits can be seen in Table S1. The TFLC amounts in the samples were determined to be 411.40 ± 8.49 mg ce/g for EEA, 389.43 ± 8.31 mg ce/g for MEA, and 555.92 ± 9.62 mg ce/g for AEA. The TAC values of EEA, MEA, and AEA were 6740.28 ± 0.0054, 6297.85 ± 0.0025, and 5806.32 ± 0.0012 mg/L, respectively.

Table S1 presents the percentage of cell viability (%) for the different aqueous extracts derived from Aronia fruits, which were employed in this investigation to examine their cytotoxic impacts on Caco‐2 adenocarcinoma cells. The samples containing EEA, MEA, and AEA at a concentration of 10 mg/mL demonstrated a significant cytotoxic impact on the cells, resulting in inhibition ratios of 41.44%, 46.77%, and 51.22%, respectively. According to the findings presented in Table S1, the IC_50_ (half‐maximal inhibitory concentration) values for EEA, MEA, and AEA were determined to be 11.65, 11.71, and 10.51 mg/mL, respectively.

The antimicrobial activity of Aronia fruits was given in Table S1. There were no observed inhibitory effects of EEA, MEA, and AEA on the growth of *Salmonella* and *E. coli* O157:H7 bacteria. The extract that exhibits the greatest inhibitory impact on the growth of *S. aureus* was EEA, whereas the extract that does not demonstrate any discernible effect was AEA. The sample of MEA exhibits the highest degree of inhibitory qualities on the bacterium *B. cereus* , with EEA and AEA following closely behind in terms of their inhibitory effects (*p* < 0.05). The sample that exhibits the strongest antimicrobial activity against *L. monocytogenes* is MEA (*p* < 0.05). Both the EEA and the AEA exerted comparable levels of inhibition influence against *L. monocytogenes* (*p* > 0.05). The samples did not exhibit any antimicrobial activity against *C. albicans* and *S. cerevisiae* .

The criteria derived from the initial phase of the study (Table 1) were assessed for superiority by experts. Information about the experts who contributed to the study is given in Table 4. In addressing the survey, experts utilized neutrosophic linguistic measurement values. The linguistic measures and their associated neutrosophic values are outlined in Table 2 within a fuzzy neutrosophic context. A series of questionnaires was distributed to the experts to get their comments and ratings based on the established criteria. Their responses facilitated the creation of a neutrosophic decision matrix, presented in Table 5, which contains each decision‐maker's relative evaluation of the criteria. Upon assessing the consistency test (referencing Table 3) for all decision matrices completed by experts, it was determined that the test results were below 0.1, signifying acceptable values (Table 6). As a result, the analysis was conducted. The TNWAA method was utilized to consolidate the three expert viewpoints in the study into a collective group judgment. Table 7 depicts this matrix. The neutrosophic synthetic value for each identified reason was computed utilizing the aggregated pairwise comparison matrix, as delineated in Equation (4). This method facilitated the incorporation of expert assessments in uncertain conditions, which is crucial for tackling intricate, multifaceted causal factors. Table 8 displays the resultant neutrosophic synthetic values.

**TABLE 4:** The information of experts.

Experts	Qualification	Current	Serves on academia
Expert #1	Food Chemistry, Food Microbiology	Lecturer Dr.	10 years
Expert #2	Functional Foods, Food Microbiology	Assistant Professor	12 years
Expert #3	Food Biotechnology, Food Chemistry	Lecturer Dr.	9 years

**TABLE 5:** Decision matrix of criteria comparison based on the AHP scale.

DM 1										
		DPPH	TPC	CUPRAC	TFLC	TAC	IC50	*S. aureus*	*B. cereus*	*L. monocytogenes*
		*C*_1_	*C*_2_	*C*_3_	*C*_4_	*C*_5_	*C*_6_	*C*_7_	*C*_8_	*C*_9_
DPPH	*C*_1_	1.00	1.00	1.00	1.00	1.00	0.11	0.17	0.17	0.17
TPC	*C*_2_	1.00	1.00	1.00	1.00	1.00	0.11	0.17	0.17	0.17
CUPRAC	*C*_3_	1.00	1.00	1.00	1.00	1.00	0.11	0.17	0.17	0.17
TFLC	*C*_4_	1.00	1.00	1.00	1.00	1.00	0.11	0.17	0.17	0.17
TAC	*C*_5_	1.00	1.00	1.00	1.00	1.00	0.11	0.17	0.17	0.17
IC50	*C*_6_	9.00	9.00	9.00	9.00	9.00	1.00	9.00	9.00	9.00
*S. aureus*	*C*_7_	6.00	6.00	6.00	6.00	6.00	0.11	1.00	1.00	1.00
*B. cereus*	*C*_8_	6.00	6.00	6.00	6.00	6.00	0.11	1.00	1.00	1.00
*L. monocytogenes*	*C*_9_	6.00	6.00	6.00	6.00	6.00	0.11	1.00	1.00	1.00

DM 2										
		DPPH	TPC	CUPRAC	TFLC	TAC	IC50	*S. aureus*	*B. cereus*	*L. monocytogenes*
		*C*_1_	*C*_2_	*C*_3_	*C*_4_	*C*_5_	*C*_6_	*C*_7_	*C*_8_	*C*_9_
DPPH	*C*_1_	1.00	1.00	1.00	1.00	1.00	0.13	0.14	0.14	0.14
TPC	*C*_2_	1.00	1.00	1.00	1.00	1.00	0.13	0.14	0.14	0.14
CUPRAC	*C*_3_	1.00	1.00	1.00	1.00	1.00	0.13	0.14	0.14	0.14
TFLC	*C*_4_	1.00	1.00	1.00	1.00	1.00	0.13	0.14	0.14	0.14
TAC	*C*_5_	1.00	1.00	1.00	1.00	1.00	0.13	0.14	0.14	0.14
IC50	*C*_6_	8.00	8.00	8.00	8.00	8.00	1.00	7.00	7.00	7.00
*S. aureus*	*C*_7_	7.00	7.00	7.00	7.00	7.00	0.14	1.00	1.00	1.00
*B. cereus*	*C*_8_	7.00	7.00	7.00	7.00	7.00	0.14	1.00	1.00	1.00
*L. monocytogenes*	*C*_9_	7.00	7.00	7.00	7.00	7.00	0.14	1.00	1.00	1.00

DM 3										
		DPPH	TPC	CUPRAC	TFLC	TAC	IC50	*S. aureus*	*B. cereus*	*L. monocytogenes*
		*C*_1_	*C*_2_	*C*_3_	*C*_4_	*C*_5_	*C*_6_	*C*_7_	*C*_8_	*C*_9_
DPPH	*C*_1_	1.00	1.00	1.00	1.00	1.00	0.13	0.13	0.13	0.13
TPC	*C*_2_	1.00	1.00	1.00	1.00	1.00	0.13	0.13	0.13	0.13
CUPRAC	*C*_3_	1.00	1.00	1.00	1.00	1.00	0.13	0.13	0.13	0.13
TFLC	*C*_4_	1.00	1.00	1.00	1.00	1.00	0.13	0.13	0.13	0.13
TAC	*C*_5_	1.00	1.00	1.00	1.00	1.00	0.13	0.13	0.13	0.13
IC50	*C*_6_	8.00	8.00	8.00	8.00	8.00	1.00	9.00	9.00	9.00
*S. aureus*	*C*_7_	8.00	8.00	8.00	8.00	8.00	0.11	1.00	1.00	1.00
*B. cereus*	*C*_8_	8.00	8.00	8.00	8.00	8.00	0.11	1.00	1.00	1.00
*L. monocytogenes*	*C*_9_	8.00	8.00	8.00	8.00	8.00	0.11	1.00	1.00	1.00

**TABLE 6:** Decision matrix of criteria comparison based on the neutrosophic AHP scale.

Codes	DM 1									DM 2									DM 3								
	*C*_1_	*C*_2_	*C*_3_	*C*_4_	*C*_5_	*C*_6_	*C*_7_	*C*_8_	*C*_9_	*C*_1_	*C*_2_	*C*_3_	*C*_4_	*C*_5_	*C*_6_	*C*_7_	*C*_8_	*C*_9_	*C*_1_	*C*_2_	*C*_3_	*C*_4_	*C*_5_	*C*_6_	*C*_7_	*C*_8_	*C*_9_
*C*_1_	1.00	1.00	1.00	1.00	1.00	9.00	5.00	5.00	5.00	1.00	1.00	1.00	1.00	1.00	7.00	6.00	6.00	6.00	1.00	1.00	1.00	1.00	1.00	7.00	7.00	7.00	7.00
	1.00	1.00	1.00	1.00	1.00	9.00	6.00	6.00	6.00	1.00	1.00	1.00	1.00	1.00	8.00	7.00	7.00	7.00	1.00	1.00	1.00	1.00	1.00	8.00	8.00	8.00	8.00
	1.00	1.00	1.00	1.00	1.00	9.00	7.00	7.00	7.00	1.00	1.00	1.00	1.00	1.00	9.00	8.00	8.00	8.00	1.00	1.00	1.00	1.00	1.00	9.00	9.00	9.00	9.00
	1.00	1.00	1.00	1.00	1.00	9.00	8.00	8.00	8.00	1.00	1.00	1.00	1.00	1.00	9.00	9.00	9.00	9.00	1.00	1.00	1.00	1.00	1.00	9.00	9.00	9.00	9.00
	0.50	0.50	0.50	0.50	0.50	1.00	0.70	0.70	0.70	0.50	0.50	0.50	0.50	0.50	0.85	0.90	0.90	0.90	0.50	0.50	0.50	0.50	0.50	0.85	0.85	0.85	0.85
	0.50	0.50	0.50	0.50	0.50	0.00	0.25	0.25	0.25	0.50	0.50	0.50	0.50	0.50	0.10	0.10	0.10	0.10	0.50	0.50	0.50	0.50	0.50	0.10	0.10	0.10	0.10
	0.50	0.50	0.50	0.50	0.50	0.00	0.30	0.30	0.30	0.50	0.50	0.50	0.50	0.50	0.15	0.10	0.10	0.10	0.50	0.50	0.50	0.50	0.50	0.15	0.15	0.15	0.15
*C*_2_	1.00	1.00	1.00	1.00	1.00	9.00	5.00	5.00	5.00	1.00	1.00	1.00	1.00	1.00	7.00	6.00	6.00	6.00	1.00	1.00	1.00	1.00	1.00	7.00	7.00	7.00	7.00
	1.00	1.00	1.00	1.00	1.00	9.00	6.00	6.00	6.00	1.00	1.00	1.00	1.00	1.00	8.00	7.00	7.00	7.00	1.00	1.00	1.00	1.00	1.00	8.00	8.00	8.00	8.00
	1.00	1.00	1.00	1.00	1.00	9.00	7.00	7.00	7.00	1.00	1.00	1.00	1.00	1.00	9.00	8.00	8.00	8.00	1.00	1.00	1.00	1.00	1.00	9.00	9.00	9.00	9.00
	1.00	1.00	1.00	1.00	1.00	9.00	8.00	8.00	8.00	1.00	1.00	1.00	1.00	1.00	9.00	9.00	9.00	9.00	1.00	1.00	1.00	1.00	1.00	9.00	9.00	9.00	9.00
	0.50	0.50	0.50	0.50	0.50	1.00	0.70	0.70	0.70	0.50	0.50	0.50	0.50	0.50	0.85	0.90	0.90	0.90	0.50	0.50	0.50	0.50	0.50	0.85	0.85	0.85	0.85
	0.50	0.50	0.50	0.50	0.50	0.00	0.25	0.25	0.25	0.50	0.50	0.50	0.50	0.50	0.10	0.10	0.10	0.10	0.50	0.50	0.50	0.50	0.50	0.10	0.10	0.10	0.10
	0.50	0.50	0.50	0.50	0.50	0.00	0.30	0.30	0.30	0.50	0.50	0.50	0.50	0.50	0.15	0.10	0.10	0.10	0.50	0.50	0.50	0.50	0.50	0.15	0.15	0.15	0.15
*C*_3_	1.00	1.00	1.00	1.00	1.00	9.00	5.00	5.00	5.00	1.00	1.00	1.00	1.00	1.00	7.00	6.00	6.00	6.00	1.00	1.00	1.00	1.00	1.00	7.00	7.00	7.00	7.00
	1.00	1.00	1.00	1.00	1.00	9.00	6.00	6.00	6.00	1.00	1.00	1.00	1.00	1.00	8.00	7.00	7.00	7.00	1.00	1.00	1.00	1.00	1.00	8.00	8.00	8.00	8.00
	1.00	1.00	1.00	1.00	1.00	9.00	7.00	7.00	7.00	1.00	1.00	1.00	1.00	1.00	9.00	8.00	8.00	8.00	1.00	1.00	1.00	1.00	1.00	9.00	9.00	9.00	9.00
	1.00	1.00	1.00	1.00	1.00	9.00	8.00	8.00	8.00	1.00	1.00	1.00	1.00	1.00	9.00	9.00	9.00	9.00	1.00	1.00	1.00	1.00	1.00	9.00	9.00	9.00	9.00
	0.50	0.50	0.50	0.50	0.50	1.00	0.70	0.70	0.70	0.50	0.50	0.50	0.50	0.50	0.85	0.90	0.90	0.90	0.50	0.50	0.50	0.50	0.50	0.85	0.85	0.85	0.85
	0.50	0.50	0.50	0.50	0.50	0.00	0.25	0.25	0.25	0.50	0.50	0.50	0.50	0.50	0.10	0.10	0.10	0.10	0.50	0.50	0.50	0.50	0.50	0.10	0.10	0.10	0.10
	0.50	0.50	0.50	0.50	0.50	0.00	0.30	0.30	0.30	0.50	0.50	0.50	0.50	0.50	0.15	0.10	0.10	0.10	0.50	0.50	0.50	0.50	0.50	0.15	0.15	0.15	0.15
*C*_4_	1.00	1.00	1.00	1.00	1.00	9.00	5.00	5.00	5.00	1.00	1.00	1.00	1.00	1.00	7.00	6.00	6.00	6.00	1.00	1.00	1.00	1.00	1.00	7.00	7.00	7.00	7.00
	1.00	1.00	1.00	1.00	1.00	9.00	6.00	6.00	6.00	1.00	1.00	1.00	1.00	1.00	8.00	7.00	7.00	7.00	1.00	1.00	1.00	1.00	1.00	8.00	8.00	8.00	8.00
	1.00	1.00	1.00	1.00	1.00	9.00	7.00	7.00	7.00	1.00	1.00	1.00	1.00	1.00	9.00	8.00	8.00	8.00	1.00	1.00	1.00	1.00	1.00	9.00	9.00	9.00	9.00
	1.00	1.00	1.00	1.00	1.00	9.00	8.00	8.00	8.00	1.00	1.00	1.00	1.00	1.00	9.00	9.00	9.00	9.00	1.00	1.00	1.00	1.00	1.00	9.00	9.00	9.00	9.00
	0.50	0.50	0.50	0.50	0.50	1.00	0.70	0.70	0.70	0.50	0.50	0.50	0.50	0.50	0.85	0.90	0.90	0.90	0.50	0.50	0.50	0.50	0.50	0.85	0.85	0.85	0.85
	0.50	0.50	0.50	0.50	0.50	0.00	0.25	0.25	0.25	0.50	0.50	0.50	0.50	0.50	0.10	0.10	0.10	0.10	0.50	0.50	0.50	0.50	0.50	0.10	0.10	0.10	0.10
	0.50	0.50	0.50	0.50	0.50	0.00	0.30	0.30	0.30	0.50	0.50	0.50	0.50	0.50	0.15	0.10	0.10	0.10	0.50	0.50	0.50	0.50	0.50	0.15	0.15	0.15	0.15
*C*_5_	1.00	1.00	1.00	1.00	1.00	9.00	5.00	5.00	5.00	1.00	1.00	1.00	1.00	1.00	7.00	6.00	6.00	6.00	1.00	1.00	1.00	1.00	1.00	7.00	7.00	7.00	7.00
	1.00	1.00	1.00	1.00	1.00	9.00	6.00	6.00	6.00	1.00	1.00	1.00	1.00	1.00	8.00	7.00	7.00	7.00	1.00	1.00	1.00	1.00	1.00	8.00	8.00	8.00	8.00
	1.00	1.00	1.00	1.00	1.00	9.00	7.00	7.00	7.00	1.00	1.00	1.00	1.00	1.00	9.00	8.00	8.00	8.00	1.00	1.00	1.00	1.00	1.00	9.00	9.00	9.00	9.00
	1.00	1.00	1.00	1.00	1.00	9.00	8.00	8.00	8.00	1.00	1.00	1.00	1.00	1.00	9.00	9.00	9.00	9.00	1.00	1.00	1.00	1.00	1.00	9.00	9.00	9.00	9.00
	0.50	0.50	0.50	0.50	0.50	1.00	0.70	0.70	0.70	0.50	0.50	0.50	0.50	0.50	0.85	0.90	0.90	0.90	0.50	0.50	0.50	0.50	0.50	0.85	0.85	0.85	0.85
	0.50	0.50	0.50	0.50	0.50	0.00	0.25	0.25	0.25	0.50	0.50	0.50	0.50	0.50	0.10	0.10	0.10	0.10	0.50	0.50	0.50	0.50	0.50	0.10	0.10	0.10	0.10
	0.50	0.50	0.50	0.50	0.50	0.00	0.30	0.30	0.30	0.50	0.50	0.50	0.50	0.50	0.15	0.10	0.10	0.10	0.50	0.50	0.50	0.50	0.50	0.15	0.15	0.15	0.15
*C*_6_	0.11	0.11	0.11	0.11	0.11	1.00	0.11	0.11	0.11	0.11	0.11	0.11	0.11	0.11	1.00	0.11	0.11	0.11	0.11	0.11	0.11	0.11	0.11	1.00	0.11	0.11	0.11
	0.11	0.11	0.11	0.11	0.11	1.00	0.11	0.11	0.11	0.11	0.11	0.11	0.11	0.11	1.00	0.13	0.13	0.13	0.11	0.11	0.11	0.11	0.11	1.00	0.13	0.13	0.13
	0.11	0.11	0.11	0.11	0.11	1.00	0.11	0.11	0.11	0.13	0.13	0.13	0.13	0.13	1.00	0.14	0.14	0.14	0.13	0.13	0.13	0.13	0.13	1.00	0.14	0.14	0.14
	0.11	0.11	0.11	0.11	0.11	1.00	0.11	0.11	0.11	0.14	0.14	0.14	0.14	0.14	1.00	0.17	0.17	0.17	0.14	0.14	0.14	0.14	0.14	1.00	0.17	0.17	0.17
	1.00	1.00	1.00	1.00	1.00	0.50	1.00	1.00	1.00	1.00	1.00	1.00	1.00	1.00	0.50	1.00	1.00	1.00	1.00	1.00	1.00	1.00	1.00	0.50	1.00	1.00	1.00
	0.00	0.00	0.00	0.00	0.00	0.50	0.00	0.00	0.00	0.00	0.00	0.00	0.00	0.00	0.50	0.00	0.00	0.00	0.00	0.00	0.00	0.00	0.00	0.50	0.00	0.00	0.00
	0.00	0.00	0.00	0.00	0.00	0.50	0.00	0.00	0.00	0.00	0.00	0.00	0.00	0.00	0.50	0.00	0.00	0.00	0.00	0.00	0.00	0.00	0.00	0.50	0.00	0.00	0.00
*C*_7_	0.13	0.13	0.13	0.13	0.13	9.00	1.00	1.00	1.00	0.11	0.11	0.11	0.11	0.11	6.00	1.00	1.00	1.00	0.11	0.11	0.11	0.11	0.11	9.00	1.00	1.00	1.00
	0.14	0.14	0.14	0.14	0.14	9.00	1.00	1.00	1.00	0.13	0.13	0.13	0.13	0.13	7.00	1.00	1.00	1.00	0.11	0.11	0.11	0.11	0.11	9.00	1.00	1.00	1.00
	0.17	0.17	0.17	0.17	0.17	9.00	1.00	1.00	1.00	0.14	0.14	0.14	0.14	0.14	8.00	1.00	1.00	1.00	0.13	0.13	0.13	0.13	0.13	9.00	1.00	1.00	1.00
	0.20	0.20	0.20	0.20	0.20	9.00	1.00	1.00	1.00	0.17	0.17	0.17	0.17	0.17	9.00	1.00	1.00	1.00	0.14	0.14	0.14	0.14	0.14	9.00	1.00	1.00	1.00
	1.00	1.00	1.00	1.00	1.00	1.00	0.50	0.50	0.50	1.00	1.00	1.00	1.00	1.00	0.90	0.50	0.50	0.50	1.00	1.00	1.00	1.00	1.00	1.00	0.50	0.50	0.50
	0.00	0.00	0.00	0.00	0.00	0.00	0.50	0.50	0.50	0.00	0.00	0.00	0.00	0.00	0.10	0.50	0.50	0.50	0.00	0.00	0.00	0.00	0.00	0.00	0.50	0.50	0.50
	0.00	0.00	0.00	0.00	0.00	0.00	0.50	0.50	0.50	0.00	0.00	0.00	0.00	0.00	0.10	0.50	0.50	0.50	0.00	0.00	0.00	0.00	0.00	0.00	0.50	0.50	0.50
*C*_8_	0.13	0.13	0.13	0.13	0.13	9.00	1.00	1.00	1.00	0.11	0.11	0.11	0.11	0.11	6.00	1.00	1.00	1.00	0.11	0.11	0.11	0.11	0.11	9.00	1.00	1.00	1.00
	0.14	0.14	0.14	0.14	0.14	9.00	1.00	1.00	1.00	0.13	0.13	0.13	0.13	0.13	7.00	1.00	1.00	1.00	0.11	0.11	0.11	0.11	0.11	9.00	1.00	1.00	1.00
	0.17	0.17	0.17	0.17	0.17	9.00	1.00	1.00	1.00	0.14	0.14	0.14	0.14	0.14	8.00	1.00	1.00	1.00	0.13	0.13	0.13	0.13	0.13	9.00	1.00	1.00	1.00
	0.20	0.20	0.20	0.20	0.20	9.00	1.00	1.00	1.00	0.17	0.17	0.17	0.17	0.17	9.00	1.00	1.00	1.00	0.14	0.14	0.14	0.14	0.14	9.00	1.00	1.00	1.00
	1.00	1.00	1.00	1.00	1.00	1.00	0.50	0.50	0.50	1.00	1.00	1.00	1.00	1.00	0.90	0.50	0.50	0.50	1.00	1.00	1.00	1.00	1.00	1.00	0.50	0.50	0.50
	0.00	0.00	0.00	0.00	0.00	0.00	0.50	0.50	0.50	0.00	0.00	0.00	0.00	0.00	0.10	0.50	0.50	0.50	0.00	0.00	0.00	0.00	0.00	0.00	0.50	0.50	0.50
	0.00	0.00	0.00	0.00	0.00	0.00	0.50	0.50	0.50	0.00	0.00	0.00	0.00	0.00	0.10	0.50	0.50	0.50	0.00	0.00	0.00	0.00	0.00	0.00	0.50	0.50	0.50
*C*_9_	0.13	0.13	0.13	0.13	0.13	9.00	1.00	1.00	1.00	0.11	0.11	0.11	0.11	0.11	6.00	1.00	1.00	1.00	0.11	0.11	0.11	0.11	0.11	9.00	1.00	1.00	1.00
	0.14	0.14	0.14	0.14	0.14	9.00	1.00	1.00	1.00	0.13	0.13	0.13	0.13	0.13	7.00	1.00	1.00	1.00	0.11	0.11	0.11	0.11	0.11	9.00	1.00	1.00	1.00
	0.17	0.17	0.17	0.17	0.17	9.00	1.00	1.00	1.00	0.14	0.14	0.14	0.14	0.14	8.00	1.00	1.00	1.00	0.13	0.13	0.13	0.13	0.13	9.00	1.00	1.00	1.00
	0.20	0.20	0.20	0.20	0.20	9.00	1.00	1.00	1.00	0.17	0.17	0.17	0.17	0.17	9.00	1.00	1.00	1.00	0.14	0.14	0.14	0.14	0.14	9.00	1.00	1.00	1.00
	1.00	1.00	1.00	1.00	1.00	1.00	0.50	0.50	0.50	1.00	1.00	1.00	1.00	1.00	0.90	0.50	0.50	0.50	1.00	1.00	1.00	1.00	1.00	1.00	0.50	0.50	0.50
	0.00	0.00	0.00	0.00	0.00	0.00	0.50	0.50	0.50	0.00	0.00	0.00	0.00	0.00	0.10	0.50	0.50	0.50	0.00	0.00	0.00	0.00	0.00	0.00	0.50	0.50	0.50
	0.00	0.00	0.00	0.00	0.00	0.00	0.50	0.50	0.50	0.00	0.00	0.00	0.00	0.00	0.10	0.50	0.50	0.50	0.00	0.00	0.00	0.00	0.00	0.00	0.50	0.50	0.50

**TABLE 7:** Aggregated matrix using the TNWAA approach.

	*C*_1_							*C*_2_							*C*_3_						
*C*_1_	1.00	1.00	1.00	1.00	0.50	0.50	0.50	1.00	1.00	1.00	1.00	0.50	0.50	0.50	1.00	1.00	1.00	1.00	0.50	0.50	0.50
*C*_2_	1.00	1.00	1.00	1.00	0.50	0.50	0.50	1.00	1.00	1.00	1.00	0.50	0.50	0.50	1.00	1.00	1.00	1.00	0.50	0.50	0.50
*C*_3_	1.00	1.00	1.00	1.00	0.50	0.50	0.50	1.00	1.00	1.00	1.00	0.50	0.50	0.50	1.00	1.00	1.00	1.00	0.50	0.50	0.50
*C*_4_	1.00	1.00	1.00	1.00	0.50	0.50	0.50	1.00	1.00	1.00	1.00	0.50	0.50	0.50	1.00	1.00	1.00	1.00	0.50	0.50	0.50
*C*_5_	1.00	1.00	1.00	1.00	0.50	0.50	0.50	1.00	1.00	1.00	1.00	0.50	0.50	0.50	1.00	1.00	1.00	1.00	0.50	0.50	0.50
*C*_6_	7.61	8.32	9.00	9.00	0.90	0.00	0.00	7.61	8.32	9.00	9.00	0.90	0.00	0.00	7.61	8.32	9.00	9.00	0.90	0.00	0.00
*C*_7_	5.94	6.95	7.96	8.65	0.81	0.14	0.17	5.94	6.95	7.96	8.65	0.81	0.14	0.17	5.94	6.95	7.96	8.65	0.81	0.14	0.17
*C*_8_	5.94	6.95	7.96	8.65	0.81	0.14	0.17	5.94	6.95	7.96	8.65	0.81	0.14	0.17	5.94	6.95	7.96	8.65	0.81	0.14	0.17
*C*_9_	5.94	6.95	7.96	8.65	0.81	0.14	0.17	5.94	6.95	7.96	8.65	0.81	0.14	0.17	5.94	6.95	7.96	8.65	0.81	0.14	0.17

	*C*_4_							*C*_5_							*C*_6_						
*C*_1_	1.00	1.00	1.00	1.00	0.50	0.50	0.50	1.00	1.00	1.00	1.00	0.50	0.50	0.50	0.11	0.11	0.12	0.13	1.00	0.00	0.00
*C*_2_	1.00	1.00	1.00	1.00	0.50	0.50	0.50	1.00	1.00	1.00	1.00	0.50	0.50	0.50	0.11	0.11	0.12	0.13	1.00	0.00	0.00
*C*_3_	1.00	1.00	1.00	1.00	0.50	0.50	0.50	1.00	1.00	1.00	1.00	0.50	0.50	0.50	0.11	0.11	0.12	0.13	1.00	0.00	0.00
*C*_4_	1.00	1.00	1.00	1.00	0.50	0.50	0.50	1.00	1.00	1.00	1.00	0.50	0.50	0.50	0.11	0.11	0.12	0.13	1.00	0.00	0.00
*C*_5_	1.00	1.00	1.00	1.00	0.50	0.50	0.50	1.00	1.00	1.00	1.00	0.50	0.50	0.50	0.11	0.11	0.12	0.13	1.00	0.00	0.00
*C*_6_	7.61	8.32	9.00	9.00	0.90	0.00	0.00	7.61	8.32	9.00	9.00	0.90	0.00	0.00	1.00	1.00	1.00	1.00	0.50	0.50	0.50
*C*_7_	5.94	6.95	7.96	8.65	0.81	0.14	0.17	5.94	6.95	7.96	8.65	0.81	0.14	0.17	0.11	0.12	0.13	0.15	1.00	0.00	0.00
*C*_8_	5.94	6.95	7.96	8.65	0.81	0.14	0.17	5.94	6.95	7.96	8.65	0.81	0.14	0.17	0.11	0.12	0.13	0.15	1.00	0.00	0.00
*C*_9_	5.94	6.95	7.96	8.65	0.81	0.14	0.17	5.94	6.95	7.96	8.65	0.81	0.14	0.17	0.11	0.12	0.13	0.15	1.00	0.00	0.00

	*C*_7_							*C*_8_							*C*_9_						
*C*_1_	0.12	0.13	0.15	0.17	1.00	0.00	0.00	0.12	0.13	0.15	0.17	1.00	0.00	0.00	0.12	0.13	0.15	0.17	1.00	0.00	0.00
*C*_2_	0.12	0.13	0.15	0.17	1.00	0.00	0.00	0.12	0.13	0.15	0.17	1.00	0.00	0.00	0.12	0.13	0.15	0.17	1.00	0.00	0.00
*C*_3_	0.12	0.13	0.15	0.17	1.00	0.00	0.00	0.12	0.13	0.15	0.17	1.00	0.00	0.00	0.12	0.13	0.15	0.17	1.00	0.00	0.00
*C*_4_	0.12	0.13	0.15	0.17	1.00	0.00	0.00	0.12	0.13	0.15	0.17	1.00	0.00	0.00	0.12	0.13	0.15	0.17	1.00	0.00	0.00
*C*_5_	0.12	0.13	0.15	0.17	1.00	0.00	0.00	0.12	0.13	0.15	0.17	1.00	0.00	0.00	0.12	0.13	0.15	0.17	1.00	0.00	0.00
*C*_6_	7.86	8.28	8.65	9.00	0.97	0.00	0.00	7.86	8.28	8.65	9.00	0.97	0.00	0.00	7.86	8.28	8.65	9.00	0.97	0.00	0.00
*C*_7_	1.00	1.00	1.00	1.00	0.50	0.50	0.50	1.00	1.00	1.00	1.00	0.50	0.50	0.50	1.00	1.00	1.00	1.00	0.50	0.50	0.50
*C*_8_	1.00	1.00	1.00	1.00	0.50	0.50	0.50	1.00	1.00	1.00	1.00	0.50	0.50	0.50	1.00	1.00	1.00	1.00	0.50	0.50	0.50
*C*_9_	1.00	1.00	1.00	1.00	0.50	0.50	0.50	1.00	1.00	1.00	1.00	0.50	0.50	0.50	1.00	1.00	1.00	1.00	0.50	0.50	0.50

**TABLE 8:** Neutrosophic synthetic values (*S*_*i*_).

*S*_1_	0.02	0.02	0.03	0.03	1.00	0.00	0.00
*S*_2_	0.02	0.02	0.03	0.03	1.00	0.00	0.00
*S*_3_	0.02	0.02	0.03	0.03	1.00	0.00	0.00
*S*_4_	0.02	0.02	0.03	0.03	1.00	0.00	0.00
*S*_5_	0.02	0.02	0.03	0.03	1.00	0.00	0.00
*S*_6_	0.26	0.30	0.35	0.39	1.00	0.00	0.00
*S*_7_	0.14	0.17	0.21	0.25	1.00	0.00	0.00
*S*_8_	0.14	0.17	0.21	0.25	1.00	0.00	0.00
*S*_9_	0.14	0.17	0.21	0.25	1.00	0.00	0.00
Total	0.78	0.91	1.10	1.28	1.00	0.00	0.00

In the last phase of the neutrosophic AHP technique, the ultimate weights for each criterion are established. Table 9 presents the ranking of the main criteria. The aggregate of the normalized crisp values is equivalent to 1, ensuring thorough prioritization across all criteria. *C*_6_ is the criterion with the most weight, although there exists a substantial disparity between it and the second‐ranked criteria, *C*_7_, *C*_8_, and *C*_9_. A notable distinction exists between *C*_6_ and the first five criteria. *C*_6_, *C*_7_, *C*_8_, and *C*_9_ are distinguished from the other criteria. These weights are based on subjective expert assessments and may differ across various evaluations conducted by experts.

**TABLE 9:** The final significance weights and ranks of evaluation criteria based on N‐AHP SVTNN weights.

Codes	Criteria name	Criteria SVTNN weights							Crisp	Normalized Crisp	Rank
*C*_1_	DPPH	0.018	0.022	0.029	0.038	1.00	0.00	0.00	0.0268	0.0250	3
*C*_2_	TPC	0.018	0.022	0.029	0.038	1.00	0.00	0.00	0.0268	0.0250	3
*C*_3_	CUPRAC	0.018	0.022	0.029	0.038	1.00	0.00	0.00	0.0268	0.0250	3
*C*_4_	TFLC	0.018	0.022	0.029	0.038	1.00	0.00	0.00	0.0268	0.0250	3
*C*_5_	TAC	0.018	0.022	0.029	0.038	1.00	0.00	0.00	0.0268	0.0250	3
*C*_6_	IC50	0.204	0.269	0.378	0.494	1.00	0.00	0.00	0.3365	0.3148	1
*C*_7_	*S. aureus*	0.107	0.151	0.226	0.314	1.00	0.00	0.00	0.1995	0.1867	2
*C*_8_	*B. cereus*	0.107	0.151	0.226	0.314	1.00	0.00	0.00	0.1995	0.1867	2
*C*_9_	*L. monocytogenes*	0.107	0.151	0.226	0.314	1.00	0.00	0.00	0.1995	0.1867	2

The TOPSIS analysis involved a comparison of three distinct extracts based on nine indicators. The values derived from the N‐AHP analysis were utilized as weights in the TOPSIS analysis. In N‐AHP, the dimension or indicator that possesses the highest priority value is identified as the indicator with the greatest weight. In the context of general evaluation, the IC50 (*C*_6_) exhibits the highest weight at 0.3148, whereas *C*_1_–*C*_5_ demonstrate the lowest weight at 0.025. Table 10 displays the initial matrix that has been generated. However, the original data cannot be used directly in the analysis without normalization. Prior to conducting the TOPSIS analysis, a normalization process was implemented to facilitate the evaluation of indicators that possess varying units. Thus, the values of each indicator are normalized to a range between 0 and 1 (Erdin and Ozkaya 2020a, 2020b). This measure also ensures that the analysis remains unaffected by outliers. The normalization process is illustrated in Formula (30). In the assessment, extracts must meet minimum values for indicator *C*_6_, while they are required to achieve maximum values for the other indicators. During the analysis using multi‐criteria decision‐making methods, necessary transformations are applied to criteria with negative effects, allowing calculations to proceed similarly to those with positive effects. In this study, the indicators in the decision matrix that negatively impact performance were transformed, and TOPSIS analysis was conducted using this newly transformed matrix. The matrix after transformation is presented in Table 11.

**TABLE 10:** TOPSIS decision matrix.

	Max	Max	Max	Max	Max	Min	Max	Max	Max
	DPPH	TPC	CUPRAC	TFLC	TAC	IC50	*S. aureus*	*B. cereus*	*L. monocytogenes*
EEA	4816.13	721.74	2486.99	411.40	6740.28	**11.65**	7.00	8.00	6.00
MEA	5907.78	934.03	2582.25	389.43	6297.85	**11.71**	6.00	9.00	10.00
AEA	5790.91	956.70	3554.07	555.92	5806.32	**10.51**	0.00	7.00	6.00

*Note:* Bold values are statistically significant.

**TABLE 11:** The transformed decision matrix.

	Max	Max	Max	Max	Max	Max	Max	Max	Max
	DPPH	TPC	CUPRAC	TFLC	TAC	IC50	*S. aureus*	*B. cereus*	*L. monocytogenes*
EEA	4816.13	721.74	2486.99	411.40	6740.28	**0.0858**	7.00	8.00	6.00
MEA	5907.78	934.03	2582.25	389.43	6297.85	**0.0854**	6.00	9.00	10.00
AEA	5790.91	956.70	3554.07	555.92	5806.32	**0.0951**	0.00	7.00	6.00

*Note:* Bold values are statistically significant.

In the second step, each element of the converted decision matrix is squared, and the resulting squares are divided by the square root of the sum of each column of these squared values, as illustrated in Formula (31). A normalized decision matrix has been derived using the calculated values presented in Table 12.

**TABLE 12:** The normalized decision matrix.

	Max	Max	Max	Max	Max	Max	Max	Max	Max
	DPPH	TPC	CUPRAC	TFLC	TAC	IC50	*S. aureus*	*B. cereus*	*L. monocytogenes*
EEA	0.00	0.00	0.00	0.132	1.00	0.045	1.00	0.50	0.00
MEA	1.00	0.904	0.0893	0.00	0.527	0.00	0.857	1.00	1.00
AEA	0.893	1.00	1.00	1.00	0.00	1.00	0.00	0.00	0.00

In the third step, the values computed in the preceding step are multiplied by the corresponding weights derived from the N‐AHP to produce the weighted normalized matrix. Table 13 presents the matrix.

**TABLE 13:** TOPSIS weighted normalized matrix.

	Max	Max	Max	Max	Max	Max	Max	Max	Max
*w*_*j*_ (weight)	0.025	0.025	0.025	0.025	0.025	0.315	0.187	0.187	0.187

	DPPH	TPC	CUPRAC	TFLC	TAC	IC50	*S. aureus*	*B. cereus*	*L. monocytogenes*
EEA	0	0	0	0.003276292	0.022161439	0.2168103	0.1417268	0.083479229	0
MEA	0.018679867	0.016789064	0.001667583	0	0.011663253	0.2282214	0.1214801	0.166958457	0.18666523
AEA	0.016680031	0.018581932	0.018679867	0.018581932	0	0	0	0	0

In the fourth step, the calculations for ideal and negative ideal distance values are performed. A portion of the matrix representing ideal distance values is presented in Table 14, while a portion of the matrix for negative ideal distance values is displayed in Table 15.

**TABLE 14:** TOPSIS ideal distance matrix.

	DPPH	TPC	CUPRAC	TFLC	TAC	IC50	*S. aureus*	*B. cereus*	*L. monocytogenes*	Total	$S_{i}^{*}$
EEA	0.000348937	0.000345288	0.000348937	0.000234263	0	0.0001302	0	0.006968782	0.034843908	0.04322033	0.20789499
MEA	0	3.21438E‐06	0.000289418	0.000345288	0.000110212	0	0.0004099	0	0	0.00115806	0.03403029
AEA	3.99935E‐06	0	0	0	0.000491129	0.052085	0.0200865	0.027875126	0.034843908	0.13538567	0.36794791

**TABLE 15:** TOPSIS negative ideal distance matrix.

	DPPH	TPC	CUPRAC	TFLC	TAC	IC50	*S. aureus*	*B. cereus*	*L. monocytogenes*	Total	$S_{i}^{-}$
EEA	0	0	0	1.07341E‐05	0.000491129	0.0470067	0.0200865	0.006968782	0	0.07456386	0.27306384
MEA	0.000348937	0.000281873	2.78083E‐06	0	0.000136031	0.052085	0.0147574	0.027875126	0.034843908	0.13033109	0.36101398
AEA	0.000278223	0.000345288	0.000348937	0.000345288	0	0	0	0	0	0.00131774	0.03630065

In the last two steps, the ideal and negative ideal values are computed, followed by the derivation of the relative proximity value for each alternative. A ranking table is generated by arranging these values in descending order, from the best alternative to the worst alternative. The values and ranking are presented in Table 16.

**TABLE 16:** TOPSIS ideal and negative ideal solution values and ranking.

Alternatives	$S_{i}^{*}$	$S_{i}^{-}$	$C_{i}^{*}$	Rank
EEA	0.2079	0.2731	0.5677	2
MEA	0.0340	0.3610	0.9139	1
AEA	0.3679	0.0363	0.0898	3

## 4 Discussion

The Aronia fruits are extensively utilized as a functional food because of their various biological properties. The abundance of polyphenols in Aronia fruits is extensively recognized. Therefore, it is imperative to extract these bioactive chemicals using suitable conditions and enhance their concentration within the extract to assess their quality effectively. Hence, variations in bioactive constituents among the extracts were assessed by employing diverse solvents.

Phenolic chemicals, which possess significant antioxidant potential, are a crucial category of bioactive compounds derived from black chokeberries (Tolic et al. 2017). The present investigation revealed that the TPC of Aronia fruits ranged from 721.74 ± 2.92 to 956.70 ± 11.65 mg GAE/g. According to a study by Engin and Mert (2020), the TPC content of Aronia fruits collected at various times ranged between 1899.70 and 1997.40 mg GAE/100 g. However, in the present investigation, the levels of TPC were found to be elevated. This phenomenon can be explained by the direct use of chokeberry juice without solvent extraction.

The antioxidant activity of anthocyanins is accountable for specific biological functions, including the prevention and reduction of the risk associated with cardiovascular diseases, cancer, and diabetes, according to the most recent research (Miguel 2011). In our study, the extract of Aronia fruits with the highest amount of anthocyanin was obtained with ethanol. Similarly, in a study where water and 80% ethanol were used as solvents, the extract with a high anthocyanin content was obtained with ethanol (Hwang and Lee 2020).

The Aronia fruit possesses a variety of nutrients and bioactive compounds, including proanthocyanidins, anthocyanins, and polyphenols. There exists a strong correlation between bioactive compounds and pharmacological activity. The compound exhibits various clinical applications, including antioxidant, anti‐aging, hypoglycemic, anti‐inflammatory, antibacterial, hepatoprotective, and anticancer effects (Lei et al. 2023). The utilization of Aronia fruit extract has been identified as a viable agent in the field of cancer therapy. The anthocyanin‐rich extracts derived from *A. melanocarpa* demonstrated antiproliferative effects on colon cancer cells (Yu et al. 2021). The other study indicates that the proliferation of HT‐29 cells was significantly suppressed by approximately 60%–70% after being exposed to a concentration of 50 μg/mL of monomeric anthocyanin for a duration of 24 h (Malik et al. 2003). Another study showed that a concentration of 0.25 mg/mL of *A. melanocarpa* exhibited a substantial inhibitory effect on the proliferation of cancer cells, resulting in a reduction of 24.7% (Gill et al. 2021). In the results of a study (Caliskan et al. 2023), the MTT assay test demonstrated that the Aronia extract exhibited a half‐maximal inhibitory concentration (IC_50_) of 186 μg/mL after 48 h of treatment in the HT‐29 cell line, resulting in 50% cell death. On the other hand, the IC_50_ value of Aronia fruit extract was observed to be greater for the Caco‐2 cell line in this investigation.

The antimicrobial properties of Aronia fruit extracts have been demonstrated, specifically against various kinds of bacteria, including *B. cereus* , *E. coli* , *P. aeruginosa* , and *S. aureus* (Shahin et al. 2019). The antimicrobial activity of Aronia fruits is thought to be attributed to anthocyanins. The antimicrobial action of anthocyanins derived from Aronia fruits has been demonstrated in scientific literature. This activity is attributed to the disruption of bacterial cell wall integrity, ultimately resulting in cell death (Deng et al. 2021). All the extracts obtained in this study did not exhibit any inhibitory effect on the growth of *E. coli* O157:H7 and *Salmonella*. Furthermore, the extracts exhibited no discernible antimicrobial impact on *C. albicans* and *S. cerevisiae* . The investigation yielded comparable findings as reported by Liepiņa et al. (2013).

The Aronia fruits are widely recognized for their high polyphenol content and remarkable antioxidant properties (Cho et al. 2023). In the present study, the antioxidant activity of Aronia fruit was determined to be substantial using both methodologies. Nevertheless, it was found that the sample extracted with methanol exhibited the greatest antioxidant activity in the DPPH method, but the sample extracted with acetone yielded the best value in the CUPRAC method. The dissimilarities observed between the two tests can be attributed to the distinct mechanisms of action employed by the DPPH and CUPRAC assays.

The evaluation of the bioactive, antimicrobial, and cytotoxic properties of extracts derived from Aronia fruit using methanol, ethanol, and acetone was conducted through ANOVA. The results for each dataset are detailed above. ANOVA results indicate that one extract excels under one criterion, while another extract does better under a different criterion. Consequently, comparing the alternatives or samples is exceedingly challenging. The N‐AHP‐based TOPSIS method was utilized as a multi‐criteria decision‐making technique to rank extracts based on their bioactive characteristics, antimicrobial activity, and cytotoxic effects of Aronia fruits. The criteria comprised in TOPSIS analysis were DPPH, CUPRAC, TAC, TPC, TFLC, IC50 values of extracts, and inhibition effects of extract on *S. aureus* , *B. cereus* , and *L. monocytogenes* . This study employed the N‐AHP‐based TOPSIS method for the comprehensive evaluation of all data. To ensure that the criteria would not have an equal impact on the ranking, the established criteria were assigned weights using the N‐AHP method based on expert evaluations. Prior to the N‐AHP weighing, the study's goal was clarified to field specialists, and a questionnaire was completed. All specialists asserted that bioactive components should uniformly influence analytical results. The IC50 value received the highest score. The antimicrobial results were intended to have an equal impact on the analysis outcomes. Upon weighting the obtained scores, it was determined that the IC50 value should have a greater influence on the ranking, followed by the results of the antimicrobial analysis and the content of bioactive substances.

In constructing the TOPSIS decision matrix, Aronia extracts are required to have a minimum value in the indicator *C*_6_, while they are expected to have maximum values for the other indicators (*C*_1_, *C*_2_, *C*_3_, *C*_4_, *C*_5_, *C*_7_, *C*_8_, and *C*_9_). This is because the Aronia extract concentration required to inhibit 50% of cells should be lower as the amount of bioactive substance in the content increases. The ranking established by the TOPSIS analysis, which incorporated weights derived from N‐AHP, is as follows: MEA > EEA > AEA. The results indicate that the extract derived from methanol extraction exhibits the most significant inhibitory effect on the Caco‐2 adenocarcinoma cells and the identified bacteria.

Consequently, in this study, several factors were weighted based on expert opinion, and the extracts were categorized by considering their differences and similarities. The extracts that yield optimal content conditions will be beneficial for the development of novel systems through evaluation in pharmacological investigations (Petrișor et al. 2023, 2024). Furthermore, it appears feasible to acquire more comprehensive information using N‐AHP‐based TOPSIS through an increase in the sample numbers.

## 5 Conclusion

The primary objective of this investigation was to assess the levels of bioactive constituents, as well as the anticancer, antimicrobial, and antifungal properties of Aronia fruit extracts using various solvents and compare the extracts according to solvent type with the help of the N‐AHP‐based TOPSIS method. Based on the results of the ANOVA, the samples of MEA and AEA had markedly greater antioxidant properties compared to the EEA sample, which aligns with the anticipated variation in polyphenol concentration. The sample denoted as AEA had the highest total flavonoid content, whereas the sample called EEA demonstrated the highest TAC. The antiproliferative effect shown on the Caco‐2 adenocarcinoma cell line by EEA, MEA, and AEA displayed a similar degree of efficacy. The antimicrobial properties of EEA, MEA, and AEA were observed to have an impact on *S. aureus* , *B. cereus* , and *L. monocytogenes* , whereas no apparent antimicrobial effect was observed on *Salmonella*, *E. coli* O157:H7, *C. albicans* , and *S. cerevisiae* . To determine the most effective solvent based on all criteria, they were weighted according to expert opinions using the N‐AHP method. Subsequent evaluation of the real data with the TOPSIS method revealed that MEA was the best solvent, while AEA was identified as the least effective solvent. The results indicate that 50% of Caco‐2 adenocarcinoma cells would be inhibited more effectively by the extract obtained through methanol extraction, which also demonstrated significant antimicrobial activity.

As a conclusion, this study employed the N‐AHP‐based TOPSIS method to assess the alternatives, utilizing criteria weightings established through expert opinion. This approach has demonstrated N‐AHP‐based TOPSIS's ability to identify balanced solutions in multi‐criteria contexts. This method will facilitate the elimination of predetermined criteria and the selection of alternatives according to expert opinion. The application of the N‐AHP‐based TOPSIS method within the realms of food and phytochemical sciences remains constrained. This study exemplifies the method's applicability in these areas, and its potential for use in future research is promising.

## Author Contributions

G.U.O. conceived the study, acquired and analyzed the data, drafted and revised the manuscript, validated the data, and supervised the study. The author read and approved the final manuscript.

## Conflicts of Interest

The author declares no conflicts of interest.

## Supporting information


**Table S1.** Bioactive properties of *Aronia melanocarpa* prepared with different extraction methods.
**Table S2.** Cytotoxic effect of different extracts of Aronia fruit on Caco‐2 adenocarcinoma cells.
**Table S3.** Antibacterial effect of different extracts of Aronia fruit.

## Data Availability

The author declares that the data supporting the findings of this study are available within the paper. Should any raw data files be needed in another format, they are available from the corresponding author upon reasonable request.
