# Supplementary material for: A Hybrid N‐AHP‐Based TOPSIS Decision Support Approach for Investigation of the Effect of Different Solvents on the Bioactive Properties, Anticancer, and Antimicrobial Activities of Aronia melanocarpa Extract
Source: Food Sci Nutr. 2025 Mar 30;13(4):e70122. doi: 10.1002/fsn3.70122 (PMC11955069; doi:10.1002/fsn3.70122)
Supplement: Supplementary file 1 — Table S1. Bioactive properties of Aronia melanocarpa prepared with different extraction methods. Table S2. Cytotoxic effect of different extracts of Aronia fruit on Caco‐2 adenocarcinoma cells. Table S3. Antibacterial effect of different extracts of Aronia fruit. [file FSN3-13-e70122-s001.docx]

Supporting information for

**A hybrid N-AHP-based TOPSIS decision support approach for investigation of the effect of different solvents on the bioactive properties, anticancer, and antimicrobial activities of *Aronia melanocarpa* extract**

Gulsum Ucak Ozkaya^a^

*^a^Mimar Sinan Art Fine University, Scientific Research Projects Coordination Unit, 34349, Istanbul, Turkey, ORCID: 0000-0002-4207-6797*

**Keywords:** *Aronia melanocarpa*, N-AHP-based TOPSIS, bioactive component, antimicrobial activities, antioxidant activities, cytotoxic activities

**Table of Contents**

Table S1. Bioactive properties of *Aronia melanocarpa* prepared with different extraction methods

Table S2. Cytotoxic effect of different extracts of Aronia fruit on Caco-2 adenocarcinoma cells

Table S3. Antibacterial effect of different extracts of Aronia fruit

Table S1. Bioactive properties of *Aronia melanocarpa* prepared with different extraction methods

|  | TPC (mg GAE/g) | Total Antioxidant Capacity (mg TE/g) | | TFLC (mg CE/g) | TAC (mg/L) |
| --- | --- | --- | --- | --- | --- |
|  |  |  |  |  |  |
|  |  | DPPH | CUPRAC |  |  |
|  |  |  |  |  |  |
| EEA | 721.74±2.92^b^ | 4816.13±18.23^c^ | 2486.99±39.08^b^ | 411.40±8.49^b^ | 6740.28±0.0054^a^ |
| MEA | 934.03±30.86^a^ | 5907.78±10.16^a^ | 2582.25±20.93^b^ | 389.43±8.31^c^ | 6297.85±0.0025^b^ |
| AEA | 956.70±11.65^a^ | 5790.91±50.37^b^ | 3554.07±18.72^a^ | 555.92±9.62^a^ | 5806.32±0.0012^c^ |
|  |  |  |  |  |  |

GAE, gallic acid equivalent; CE, catechin equivalent; TE, Trolox equivalent; DPPH, 1,1-diphenyl-2-picrylhydrazyl; CUPRAC, copper (II)-reducing antioxidant capacity. Data are means ± standard deviations of triplicate determinations (n=3). Comparison is between values in rows, meaning with the same letter are not significantly different (*p>0.05*).

Table S2. Cytotoxic effect of different extracts of Aronia fruit on Caco-2 adenocarcinoma cells

| Concentrations | Caco-2 cell viability (%) | | |
| --- | --- | --- | --- |
|  | EEA | MEA | AEA |
|  |  |  |  |
| 10 mg/mL | 58.86±0.059 | 53.23±0.026 | 48.78±0.029 |
| 8 mg/mL | 63.03±0.144 | 67.89±0.062 | 65.53±0.004 |
| 6 mg/mL | 87.83±0.073 | 78.94±0.064 | 84.98±0.047 |
| 4 mg/mL | 93.60±0.128 | 86.79±0.019 | 100.90±0.112 |
| 2 mg/mL | 100.62±0.052 | 87.14±0.022 | 105.27±0.060 |
| 1 mg/mL | 102.77±0.071 | 95.48±0.064 | 110.00±0.029 |
| 0.5 mg/mL | 110.56±0.059 | 99.72±0.033 | 113.13±0.093 |
|  |  |  |  |

Table S3. Antibacterial effect of different extracts of Aronia fruit

|  | Samples | | |
| --- | --- | --- | --- |
| Microorganisms | EEA | MEA | AEA |
|  |  |  |  |
| *Salmonella* Typhimurium | *NZ | NZ | NZ |
| *Staphylococcus aureus* | 7.00±0.00^a^ | 6.00±0.00^b^ | NZ |
| *Escherichia coli* O157:H7 | NZ | NZ | NZ |
| *Bacillus cereus* | 8.00±0.00^b^ | 9.00±0.00^a^ | 7.00±0.00^c^ |
| *Listeria monocytogenes* | 6.00±0.00^b^ | 10.0±0.00^a^ | 6.00±0.00^b^ |
|  |  |  |  |

The measurement of inhibition zones is expressed in millimeters (mm).*NZ: No zone. A notable distinction exists among means that are represented by distinct letters within the same row (p< 0.05).
